# A workflow for the relative quantification of multiple fish species from oceanic water samples using environmental DNA (eDNA) to support large-scale fishery surveys

**DOI:** 10.1371/journal.pone.0257773

**Published:** 2021-09-27

**Authors:** Ana Ramón-Laca, Abigail Wells, Linda Park

**Affiliations:** 1 School of Marine Environmental Affairs, University of Washington, Seattle, WA, United States of America; 2 Northwest Fisheries Science Center, National Oceanic and Atmospheric Administration, Seattle, WA, United States of America; 3 Lynker Technologies, Leesburg, VA, United States of America; University of Hyogo, JAPAN

## Abstract

While the number of published marine studies using environmental DNA (eDNA) has increased substantially in recent years, marine fish surveys are still scarce. To examine the potential for eDNA to support marine fisheries monitoring surveys, we optimized an eDNA isolation method, developed a multispecies assay and tested it on eDNA samples collected along the Pacific coast of the United States. Four commercial DNA extraction kits that exploit the capability of the nucleic acids binding a solid phase (two using a silica matrix and two magnetic beads) as well an organic separation method were tested. A species-specific multiplex qPCR assay was developed and tested to simultaneously target Pacific hake (*Merluccius productus*), Pacific lamprey (*Entosphenus tridentatus*) and eulachon (*Thaleichthys pacificus*). The specificity of the assay was tested in silico, in vitro and in natura. Environmental DNA isolation using phenol:chloroform:isoamyl purification with a phase lock was optimized and yielded the highest amount of total and target DNA and was used to extract 46 marine water samples for the detection of the three species of interest. The multiplex qPCR assay used in the quantification process was also optimized to provide convenience and accuracy. Pacific hake was present in 44% of the eDNA samples while the other two species were absent. Here, we present a complete workflow for the simultaneous detection and quantification of multiple marine fish species using eDNA. This workflow supports large-scale at-sea sampling efforts with preservation at ambient temperatures and has demonstrated DNA extraction efficiency and reliability. The multiplex qPCR assay is shown to be sensitive and specific for the purposes of simultaneously monitoring the relative abundance of multiple targeted fish species.

## Introduction

The use of environmental DNA (eDNA) from water samples either to detect specific targets or characterize community composition is becoming a regular monitoring tool used in aquatic studies. However, despite the increasing use of this non-invasive tool and demonstrated correlations between species-specific DNA concentrations and biomass and abundance of the species [1–3], quantification of eDNA for monitoring the abundance of marine fish for management purposes is still in its infancy (a review in Hansen, Bekkevold [4]). There are a number of studies comparing metabarcoding data to trawls [3,5] and targeted eDNA quantification, in some cases compared to trawls or acoustic data [6–8]. To support the goals of marine fisheries monitoring, robust approaches for all stages of the workflow, from eDNA collection, through extraction and target quantification are required.

This study endeavored to develop a complete workflow from sampling for marine fish eDNA to the development of a quantitative assay that is reliable, sensitive and reproducible and can subsequently be used to conduct accurate and consistent long-term monitoring and relative quantitative assays for targeted fish species. The lack of previous oceanic eDNA validation efforts for fisheries assessment, the limited pilot studies available with real life samples, together with the constraints imposed by large-scale at-sea sampling, compelled the evaluation of the efficiency at each step of the eDNA analysis and the validation of the combination of methods used in this study. Hence, the following parameters were considered: water filtration volume, filter pore size, sample preservation, DNA extraction, quantification (qPCR) optimization and PCR inhibition.

The fishery of *Merluccius productus* (Pacific hake or whiting; Ayres, 1855) is the largest of the West coast of the United States (U.S.) in terms of landings. Fisheries surveys, such as the biennial Joint U.S.-Canada Integrated Ecosystem and Pacific Hake Acoustic Trawl Survey estimate the stocks of *M*. *productus*. The expectation of eDNA potentially augmenting metrics of fish abundance comes with logistical challenges. Constraints on marine surveys such as the above include long periods of time at sea, limited storage capacity, shipboard restrictions on hazardous chemicals and/or limited temperature-controlled sample storage options and the detectability of the species of interest.

The duration and extent of fishery monitoring cruises necessitates the shipboard filtration of sampled water for eDNA to avoid DNA degradation and storage of vast numbers and volumes of samples. To maximize eDNA capture, large volumes of water should be collected. High-sampling surveys with limited staffing warrant a filter pore size that increases capture of eukaryotic cells with minimal clogging and filtration time. Restrictions on hazardous chemicals aboard marine vessels, and transport of supplies and reagents including across state lines within the US, require hazardous or regulated reagents (e.g. ethanol) be minimized. The use of Longmire’s lysis buffer, an unrestricted sample preservation buffer [9] useable at ambient temperature for long periods of time has been characterized and established as an eDNA preservation method [10,11] and it has successfully been used to collect eDNA samples on different vessels with varying storage capabilities [12,13].

DNA yield is critical for the purposes of species-specific quantification on targeted assays compared to metabarcoding studies. While numerous eDNA studies have reported good results with commercial kits [14], often, organic extraction generates the highest DNA yield. Based on the results of Shahraki et al [15] and the simplicity of magnetic beads-based methods, the organic DNA isolation method was compared against three commercially available solid phase DNA isolation methods that use paramagnetic beads. In addition, a subset of replicate filters was stored frozen to test a widely used and purposely designed commercial kit for the isolation of genomic DNA from filtered water [16–18]. While freezer storage is not always available on vessels, this storage method was included to evaluate its relative performance and potential for use when storage allows. The preferred storage/extraction method should maximize the amount of DNA recovered while avoiding inhibitors to avoid false negative results.

Environmental DNA can be used to infer the relative abundance of fish by developing species-specific primers and hydrolysis probes using quantitative PCR or qPCR [6,19,20]. The ultimate goal of this study was to develop an optimized qPCR protocol to be used to quantify commercially important species during marine surveys. The main target species chosen to test the methods was *Merluccius productus*. In order to maximize the information obtained from the samples collected with minimal additional effort, a multi-species qPCR assay can be used. To test the ease with which a multiplex PCR could be developed, two endangered, anadromous fish species often found in conjunction with *M*. *productus* along the Pacific Coast of the United States (U.S.) were also targeted: *Entosphenus tridentatus* (Pacific lamprey; Richardson, 1836) and *Thaleichthys pacificus* (eulachon; Richardson, 1836). *Entosphenus tridentatus* is a species of concern and in decline or extirpated in many areas of Northwest of the U.S. [21]. Little is known about the migration patterns of the oceanic life stage of *Thaleichthys pacificus* and the southern distinct population segment is classified as threatened under the Endangered Species Act (U.S.) [22].

Here, an optimized eDNA sampling and laboratory assay protocol for use in marine fisheries assessment is characterized. Five DNA isolation methods were tested to maximize the recovery of target species DNA and a sensitive, specific, and accurate multispecies quantitative assay was developed.

## Materials and methods

The National Oceanographic and Atmospheric Administration (NOAA) Fisheries and Fisheries and Oceans Canada (DFO) undertake biennial coastwide Integrated ecosystem and Pacific hake acoustic and trawl surveys from Point Conception in California (United States) to Dixon Entrance in the north of British Columbia (Canada). Typically, east-west transects (ranging from 50–150 m isobath) are performed every 10 nautical miles over a three-month period of time (map in S1 File). Sample collection methods were approved by NOAA, NMFS.

### Sample collection

Sea water samples were collected aboard the NOAA ship Bell M. Shimada during the course of an Integrated ecosystem and Pacific hake acoustic and trawl survey in the summer of 2019. Among the 1873 samples collected, 20 were collected off the coast of Cape Flattery, Washington (US) (48 23.88 N, 125 28.39 W) specifically for methods evaluation, hereafter ‘extraction test samples’. A CTD rosette with ten Niskin bottles (10 L) was deployed at a depth of 120 m at a site where a mixed aggregation of fish had been observed with the acoustic echo-sounders and caught by trawling at a depth of 103 m over a 175 m bottom. All ten Niskin bottles were triggered simultaneously and two replicate samples of 2.5 L of seawater were collected on deck from each of the bottles (n = 20). The rest of the samples from the survey (‘survey samples’, n = 1853) were collected along the voyage transects at varying depths, triggering two Niskin bottles on the rosette simultaneously at each sampling depth. One sample of 2.5 L was collected from each of these Niskin bottles. Negative sampling controls were collected routinely by filtering 2.5 L of distilled water from either the onboard evaporator (in the engine room) or from jugs of distilled water brought from the laboratory for this purpose. Three of the survey samples were used to test an organic extraction phase lock method -hereafter ‘phase lock subset’- and 43 survey samples plus 10 sampling negative controls -hereafter ‘validation subset’- were used for final assay assessment (Table 1 and ‘samples details & qPCR results’ in S2 File). The rest of the survey samples were beyond the scope of this analysis and will be analyzed in a future publication.

**Table 1:** Samples analyzed in this study summarized by sample purpose (type of sample), station location (latitude, longitude), and number of samples by depth.

type of sample	Station location	Surface	25-50m	>120m
extraction	85–0 (48.4,-125.47)	-	-	20
phase lock	69–4 (45.73,-124.77)	-	-	1
phase lock	71–4 (46.06,-124.84)	-	-	2
validation	26–2 (38.56,-123.66)	2	2	-
validation	26–3 (38.56,-123.98)	2	-	-
validation	35–1 (40.07,-124.14)	2	4	-
validation	35–7 (40.06,-124.64)	1	2	-
validation	39–1 (40.73,-124.37)	2	2	-
validation	39–5 (40.73,-124.66)	2	-	-
validation	39–9 (40.73,-125.1)	-	2	-
validation	53–3 (43.06,-124.87)	1	-	-
validation	53–7 (43.07,-125.25)	2	2	-
validation	60–2 (44.23,-124.54)	-	2	-
validation	60–4 (44.23,-125.16)	2	2	-
validation	67–1 (45.4,-124.04)	2	-	-
validation	67–3 (45.4,-124.42)	1	2	-
validation	77–2 (47.06,-124.87)	2	-	-
validation	81–9 (47.73,-125.74)	-	2	-

All water samples (extraction and both subsets in survey samples) were immediately filtered using reusable 500 ml filter cups (KP-47W, Advantec®, Japan) to reduce storage volume of supplies and subsequent waste (normally burned at sea or packed back to shore). The cups were fit with 47 mm diameter mixed cellulose ester (MCE) sterile filters (composed of a combination of cellulose nitrate and cellulose acetate) with a 1 μm pore size (Advantec®, Japan) and supported on a triple-port manifold attached to a 1/8 HP portable diaphragm vacuum pump at -8 to -12 Hg (Gast, Benton Harbor, MI, U.S). Four filters from the extraction test subset were stored dry and frozen (-80°C) in individual, single use Whirl-Pak (Whirl-Pak, Madison, WI, U.S.) bags and 16 samples in the extraction test sample set and all survey samples were submerged in 2 ml of lysis buffer [9] in 5 mL LoBind Eppendorf (Eppendorf, Hamburg, Germany) tubes and stored at room temperature. The filter cups and forceps used to handle filters were washed with 0.5% sodium hypochlorite and rinsed in distilled water obtained from the onboard evaporator before assemblage. The benches and other laboratory surfaces, as well as the spigots of the Niskin bottles were also wiped with sodium hyplochlorite prior to sample collection. Gloves were changed frequently and a fresh pair was always used when assembling filter cups (S3 File).

### DNA isolation

Environmental DNA (eDNA) extractions and quantitative polymerase chain reaction (qPCR) preparations were carried out one month post-collection in an amplicon-free laboratory dedicated to low copy DNA research. Negative extraction controls (Longmire’s buffer) were included daily. Four of the 16 filters preserved in lysis buffer from the extraction test subset were arbitrarily assigned to each of the following procedures: 1) organic DNA isolation (i.e., phenol:chloroform:isoamyl alcohol purification, hereafter ‘PCI’, modified from [23], 2) MagAttract PowerWater DNA/RNA (Qiagen, Hilden, Germany), hereafter ‘MA-PW’, 3) Agencourt DNAdvance, hereafter ‘ADV’ and 4) Agencourt GenFind v.3 (Beckman Coulter Pasadena, CA, US), hereafter ‘AGF’ (details and modifications of each method in supporting S1 File). The four frozen filters were extracted using the DNeasy PowerWater kit (Qiagen, Hilden, Germany), hereafter ‘PW’. The corresponding sampling negative control was extracted using the AGF method. Regardless of extraction protocol, DNA was eluted in a final volume 100 μl of Tris-lowEDTA (TlowE). Low retention tubes and plates were used at all steps. DNA quality for all 20 samples in the extraction test was evaluated on a TapeStation 2200 system (Agilent, Santa Clara, CA, US). The total amount of DNA from all 20 samples and the sampling negative control was quantified using a Qubit® 3.0 fluorometer (ThermoFisher Scientific, Waltham, MA, U.S.).

To increase the throughput of the PCI DNA extraction and decrease repetitive pipetting for the anticipated 1800+ samples collected, the use of silicone vacuum grease (Dow Corning, Midland, MI, US) as a phase lock [24] between the organic and aqueous phases was evaluated (see S1 File) with the phase-lock subset of samples. The phase lock creates a stable interphase layer allowing easy decanting of the aqueous layer into the next extraction step.

**Fig 1:**
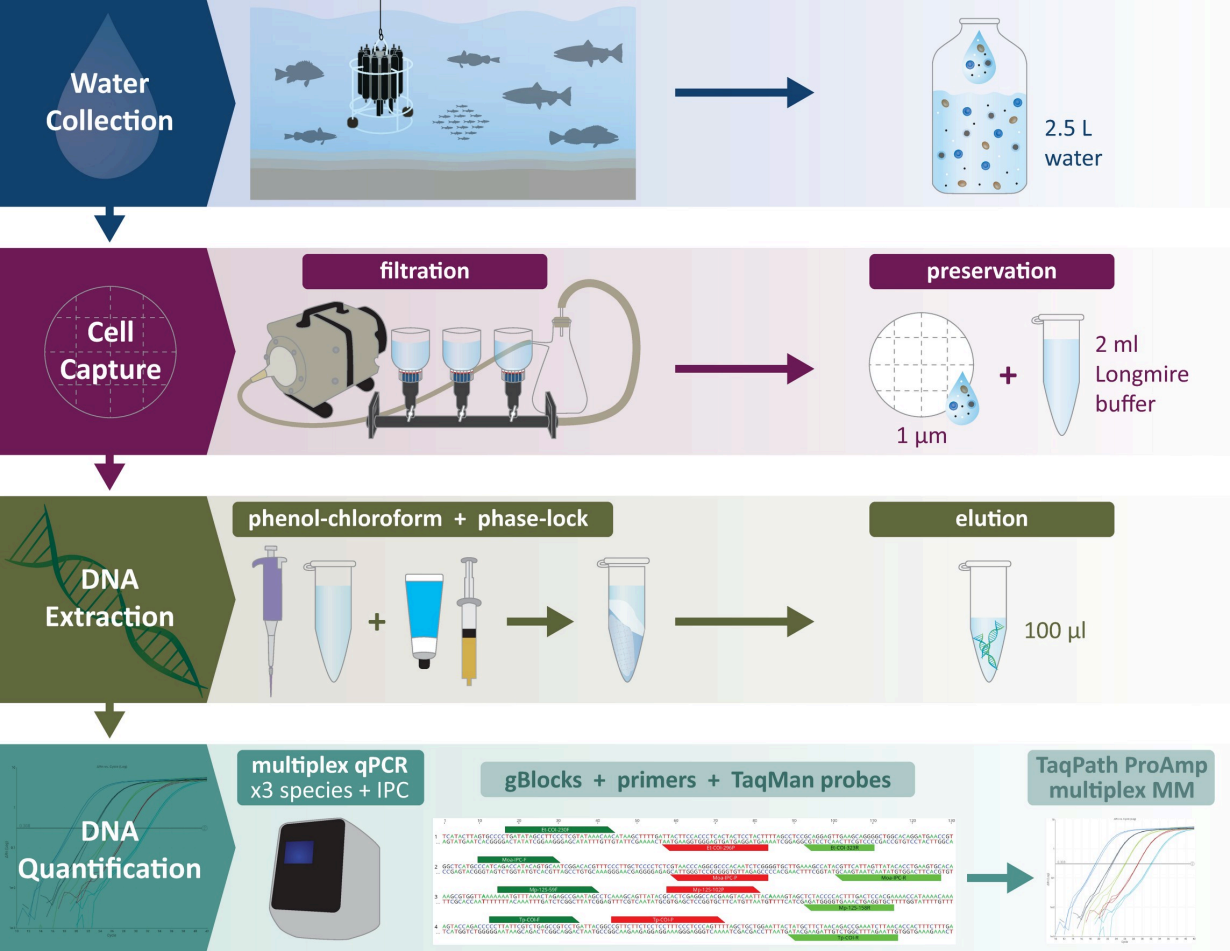
Final workflow proposed after methods evaluation. Water samples are collected at different depths using Niskin bottles on a CTD rosette and filtered immediately after. The filters are preserved in tubes with Longmire’s buffer and the DNA is extracted using a phase-lock/phenol-chloroform extraction. Target DNA is analyzed in a multiplex qPCR assay (details in S3 File). Illustration by Su Kim, NWFSC/NOAA Fisheries.

The validation subset of samples was extracted using the PCI method with the phase lock modification (Fig 1). In order to avoid any co-precipitation of salts, the final PCI method was slightly modified by conducting the precipitation with isopropanol at room temperature and adding a second 70% ethanol wash (final DNA extraction method in S1 and S3 Files; additional experiments in S1 and S2 Files).

DNA from reference samples of *M*. *productus*, *T*. *pacificus* and *E*. *tridentatus* and related species used for the specificity tests was isolated in a separate laboratory using a DNeasy Blood and Tissue Kit extraction kit (Qiagen, Hilden, Germany) and eluted in 200 μl of TlowE.

### Species-specific assay

Species-specific primers and hydrolysis probes for the cytochrome oxidase subunit 1 (COI) and 12S rRNA (12S) genes that targeted the three species of interest–*Merluccius productus*, *Entosphenus tridentatus* and *Thaleichthys pacificus* were developed (details in Table 2 and S1 File). Specificity was accounted for in silico using primerTree [25], an R package that uses the Primer BLAST search [26] against all sequences available in GenBank® [27]. Results were filtered to narrow down to one or zero mismatches in each primer (S1 File). Non-target species found to match each primer set using the *primerTree* in silico specificity test were verified as either extinct or not co-occurring with the species of interest (S1 File), the latter being the case of the *Merluccius gayi* (Peruvian hake) and the *Entosphenus similis* (Klamath river lamprey). The probes themselves conferred additional specificity (S2 File). Reporter dyes on the hydrolysis probes were selected for optimal multiplex performance on an Applied Biosystems QuantStudio 6 Flex Real-Time system (ThermoFisher Scientific, Waltham, MA, US).

**Table 2:** List of species-specific primers, hydrolysis probes and targeted fragment (gBlocks) of the multiplex qPCR assay.

Common name *Species*	Amplicon Length (bp)	Oligonucleotide type	Oligonucleotide name; sequence (5’ - 3’)
Pacific lamprey *Entosphenus tridentatus*	94	forward	Et-COI-230F; TGATATAGCCTTCCCTCGTATAAACAAC
		probe	Et-COI-296P; JUN-AAGTAGGAGTAGTGAGGGTGGAAGTAA-QSY
		reverse	Et-COI-323R; CCCTGCTTCAACTCCTGC
Pacific hake *Merluccius productus*	101	forward	Mp-12S-59F; AAATGTTTAAACTAGAGCCGAATAGC
		probe	Mp-12S-102P; 6FAM-CACTCGAGGCCACGAAGTACAATT-(MGB)NFQ
		reverse	Mp-12S-158R; TCGTGGAGTCAAAGTGGGGTAGA
Eulachon *Thaleichthys pacificus*	104	forward	Tp-COI-F; CCTTATTCGTCTGAGCCGTCCTG
		probe	Tp-COI-P2; ABY-GGCCGTTCTTCTCCTCCTTTCCCTCCCAGTTT-QSY
		reverse	Tp-COI-R; GTTAAGATTTCGGTCTGTTAGAAGCATA
Broad-billed moa *Euryapteryx curtus*	118	forward	Moa-IPC-F; CCATCAGACCATACAGTGCAA
		probe	Moa-IPC-P; VIC-CCGAGATTGTGGGCGCCTGGGTTAC (MGB)NFQ
		reverse	Moa-IPC-R; GCACTTCAGGTGTATAACTAATGAACG
**gBlocks** ***Species***	**Sequence (5’-3’)**		
*Entosphenus tridentatus*	TCATACTTAGTGCCCCTGATATAGCCTTCCCTCGTATAAACAACATAAGCTTTTGATTACTTCCACCCTCACTACTCCTACTTTTAGCCTCCGCAGGAGTTGAAGCAGGGGCTGGCACAGGATGAACCGT		
*Merluccius productus*	AAGCGTGGTTAAAAAAATGTTTAAACTAGAGCCGAATAGCCTCAAAGCAGTTATACGCACTCGAGGCCACGAAGTACAATTACAAAAGTAGCTCTACCCCACTTTGACTCCACGAAAACCATAAAACAAA		
*Thaleichthys pacificus*	AGTACCAGACCCCCTTATTCGTCTGAGCCGTCCTGATTACGGCCGTTCTTCTCCTCCTTTCCCTCCCAGTTTTAGCTGCTGGAATTACTATGCTTCTAACAGACCGAAATCTTAACACCACTTTCTTTGA		
*Euryapteryx curtus*	GGCTCATGCCCATCAGACCATACAGTGCAATCGGACACGTTTCCCTTGCTCCCCTCTCGTAACCCAGGCGCCCACAATCTCGGGGTGCTTGAAAGCCATACGTTCATTAGTTATACACCTGAAGTGCACA		

Probes include the 5’and 3’modifications as used in the qPCR assay described here.

Target DNA from the extraction test subset and the sampling negative control (distilled water) was quantified using a multiplex TaqMan assay. All amplifications were carried out in triplicate in 10 μl reactions containing 1 × TaqMan^®^ Environmental Master Mix 2.0 (ThermoFisher Scientific, Waltham, MA US), forward and reverse primers (0.9 μM), hydrolysis probes (0.2 μM), and 2 μl of genomic DNA standard or eDNA. Cycling conditions were: initial denaturation step at 95°C for 10 min, followed by 45 cycles of 95°C for 15 s and 60°C for 1 min. Seven 10-fold serial dilutions starting at ca. 1 ng μl^-1^ were used to build the standard curve. The standard curves starting points were quantified on each run using a Qubit dsDNA HS kit on the Qubit® 3.0 fluorometer and ranged from 98–103 pg μl^-1^. The amplicons were sequenced on a 3500xL Genetic Analyzer using a BigDye terminator v3.1 cycle sequencing kit (ThermoFisher Scientific, Waltham, MA, US) to confirm that the signal obtained in the multiplex TaqMan assay was from the target species.

### Inhibition testing

To expand the multiplexing capabilities and minimize PCR inhibition, all qPCR tests following the initial development of the assays were performed as above using the TaqPath ProAmp Multiplex Master Mix (ThermoFisher Scientific, Waltham, MA, U.S.) (see S1 File). A synthetic, linear, double-stranded oligonucleotide gBlocks® Gene Fragments, herein ‘gBlocks’ (Integrated DNA Technologies, Coralville, IA, US), of 130 bp was synthesized from the D-loop sequence (GenBank Accession GU139002) of the extinct *Euryapteryx curtus* (broad-billed moa) as an exogenous internal positive control (IPC) of the amplification efficiency. A set of primers and a probe (Table 2) were designed and synthesized for the IPC to amplify a fragment slightly longer than those of the target species to avoid a greater efficiency than the three fish species-specific assays [28]. The purpose of the IPC was to assess amplification inhibition rates without affecting the amplification of the target, thus avoiding false negative results (absence) of the species of interest. The inhibition cut-off value was set arbitrarily as the difference between the sample IPC and the non-template control average IPC values by rounding up the deviation found among the non-template control IPC replicates (i.e., ΔCq = mean C_q_ eDNA sample—mean C_q ntc_; inhibition = ΔC_q_ > ∂C_q ntc_). Samples for which the IPC failed to amplify were given an arbitrary value of 45 as if they had amplified in the last cycle.

### Standards multiplexing validation

To enhance accuracy in the quantification and simplify the qPCR set up, gBlocks were also manufactured to provide standards for the target species [29]. The gBlocks comprise specific 130 base pairs (bp) fragments of the genes 12S for *M*. *productus* and COI for *T*. *pacificus* and *E*. *tridentatus* (S1 File). The performance of the standards from genomic DNA and the gBlocks were compared in the multiplex reaction and adding 1 μl of the IPC at a medium concentration range (1000 copies μl^-1^) in the master mix [30]. To build a standard curve, six 10-fold dilutions of the gBlocks were included starting from 10^5^ copies down to 1 copy. In addition, *M*. *productus*-specific gBlocks were run in a specific singleplex to validate performance in the multiplex reaction individually and combined with the gBlocks of the two other species. Hereafter, target DNA concentration is expressed in number of copies per microliter.

### Validation of the triple species-specific assay

The multiplex qPCR assay was examined in vitro for robustness, accuracy and specificity by testing genomic DNA from multiple individuals for each species, including 32 *M*. *productus* collected along the US West Coast and seven from the Salish Sea (described as two discrete populations), 33 *E*. *tridentatus* from along the coast of Washington and Oregon (U.S), and 45 *T*. *pacificus* samples (five individuals from nine different populations) ranging from northern British Columbia (Canada) to northern California (U.S.). The specificity was also tested by testing 13 DNA samples of four species in the order Gadiformes and one *Lampetra ayresii* (Western River lamprey) (Table 3). The DNA samples were run in duplicate at ca. 3 ng μl^-1^ and the combined gBlocks standards in triplicate. To corroborate the specificity of the assay the sequences of dubious PCR products were sequenced on a 3500xL Genetic Analyzer.

**Table 3:** List of species used for the specificity and reproducibility tests and number of positive results in the multiplex assay.

			Positive detection per species		
Species	Sample origin	n	Pacific hake	Pacific lamprey	eulachon
Pacific lamprey *Entosphenus tridentatus*	WA & OR	34	13 (2*)	34	
Western river lamprey *Lampetra ayresii*		1		1**	
Eulachon *Thaleichthys pacificus*	BC, WA, OR, CA (9 rivers)	45		13 (4*)	45
Pacific hake *Merluccius productus*	PS, WA, OR, CA	40	40		
Alaska pollock *Gadus chalcogrammus*		3	3 (3*)		
Pacific cod *Gadus macrocephalus*		4	1 (0*)		
Pacific tomcod *Microgadus proximus*		4			1 (0*)
Arctic cod *Boreogadus saida*		1			

Sample origin: Regions where the samples were collected, BC: British Columbia (Canada), PS: Puget Sound, WA: Washington, OR: Oregon, CA: California (United States); n: Number of specimens included in the test.

(*) Samples that showed amplification at > 1 copy μl^-1^ out of the total number of samples that showed amplification

** 81 copies μl^-1^ of Pacific lamprey were found in a 40 ng μl^-1^ DNA sample of Western River lamprey.

The validation subset of samples was quantified using the multiplex assay. Of the 43 survey eDNA samples, 75% were selected from sites or depths with high levels of chlorophyll a fluorescence (CTD readings>1 μg L^-1^) to evaluate whether chlorophyll a (common in surface water samples) could directly or indirectly inhibit the amplification. Samples exhibiting signs of qPCR inhibition were purified using a OneStep™ PCR Inhibitor Removal Kit (Zymo Research, Irvine, CA, U.S.) removal column and the qPCR was repeated.

## Results

### DNA isolation methods comparison

**Fig 2:**
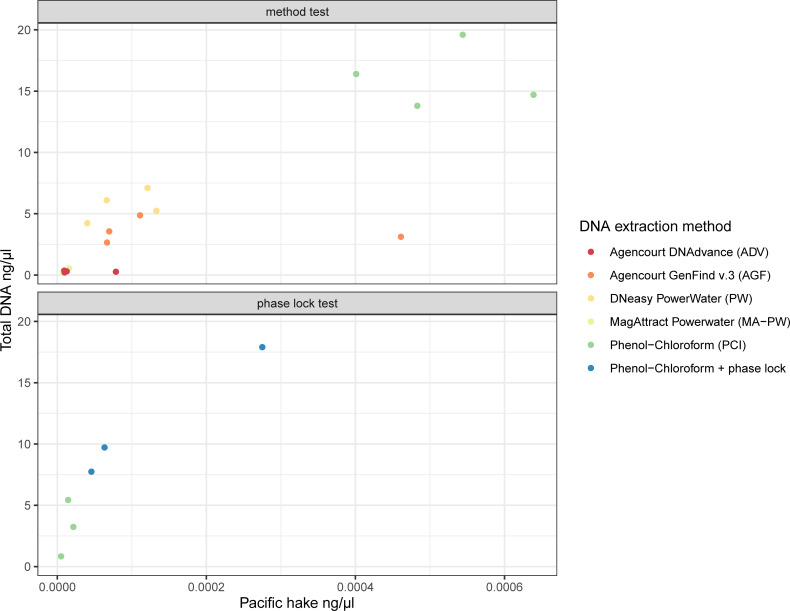
Comparison of total and target DNA quantification for five different DNA extraction methods (n = 20) and for the phenol-chloroform method with and without the phase lock with a different set of samples (n = 6, three filters split in halves). [Total DNA] was measured on the Qubit; [Merluccius productus] was measured by qPCR.

All negative controls from the DNA extraction methods comparison were negative for total and target DNA quantification. Total and target DNA was measured in each sample tested from the extraction test subset (Fig 2). The average of total DNA isolated ranged from 0.29 ng μl^-1^ using ADV to 16.13 ng μl^-1^ obtained from PCI extractions (Fig 2 and S2 File). Target DNA (*M*. *productus*) ranged from 1.03 × 10^−5^ ng μl^-1^ in the case of MA-PW to 5.17 × 10^−4^ ng μl^-1^ for PCI extraction. Two extraction methods (MA-PW and ADV) showed mean C_q_ values for *M*. *productus* beyond the lowest detectable standard, set as the limit of detection (LOD) (graph and results in S1 and S2 Files, respectively). The other three methods yielded detectable amounts of *M*. *productus* DNA. The C_q_ standard deviation of the three PCR replicates varied from 0.28 for PCI method to 1.66 for ADV (Figure in S1 File). The integrity of the DNA was variable between methods when visualized on the TapeStation (S1 File), with MA-PW and ADV being undetectable. PW generated smaller DNA fragments, most likely due to the mechanical lysis process. Regardless of the DNA extraction method, the ratio of *M*. *productus* DNA present was ca. 0.001% of the total DNA. Resulting sequences from the amplicons matched *M*. *productus*. In the phase lock subset of samples, the addition of the phase lock to the PCI extraction method yielded both more total and target DNA (Fig 2); this result was also supported by supplemental experiments in S1 and S2 Files.

### Specificity and reproducibility

**Fig 3:**
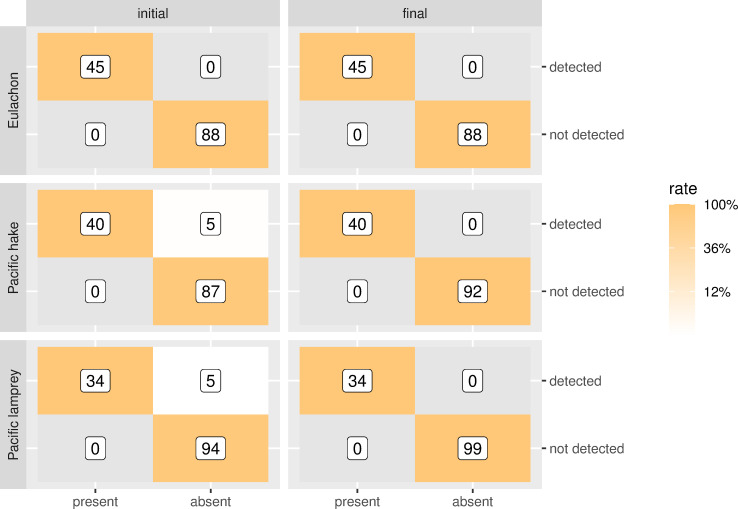
In vitro specificity results. Initial tests show the results of the qPCR with contaminated samples and final specificity is ascertained after sequencing the resulting amplicons. Color of the tiles shows the rate of the true (orange) or false positive or negative observation (light yellow). Numbers in the labels in the tiles are the number of samples accounted for in the observation at > 1 copy μl^-1^.

None of the non-template controls in the qPCR reactions showed amplification. All reference samples (*M*. *productus* n = 40, *T*. *pacificus* n = 45 and *E*. *tridentatus* n = 34) from the in vitro validation showed amplification with their respective probes (Table 3 and Fig 3). However, there was *M*. *productus* signal at low levels on 13 of 34 *E*. *tridentatus* samples with only two being at more than 1 copy μl^-1^. The *M*. *productus* primer-probe set does not amplify *Boreogadus saida* (Arctic cod), Microgradus proximus (Pacific tomcod), *Gadus chalcogrammus* (Alaska pollock), and Gadus *macrocephalus* (Pacific cod). Contamination of reference fish tissues was inferred, which most likely occurred at the time of collection for samples that were collected concurrently. However, the majority of these non-target observations were < 1 copy μl^-1^ (Table 3). More specifically, *M*. *productus* signal was obtained in one *G*. *macrocephalus* and three *G*. *chalcogrammus* samples. These were confirmed via Sanger sequencing of the amplicon as *M*. *productus* sequences (trimmed sequences of ≥25 bp were attained and matched *M*. *productus* with a 100% identity) as opposed to a false positive reaction of the assay to *G*. *chalcogrammus* or *macrocephalus* DNA. One *M*. *proximus* out of the four also showed some amplification for *T*. *pacificus* that was identified as such after sequencing, attributable to insufficient sterility measures at fishing trawl collection (see Discussion). All *T*. *pacificus* samples from Klamath and Sandy rivers and two from Elwha River showed amplification for lamprey that was found to be *E*. *tridentatus* contamination, attributable to co-occurrence in high numbers in those specific rivers (Gustafson pers. comm.). Hence, false positive and true negative rates initially close to 0 and 1 are fixed when the amplicon originated in the qPCR was sequenced (Fig 3 and S2 File).

### Validation of the triple species-specific assay

**Fig 4:**
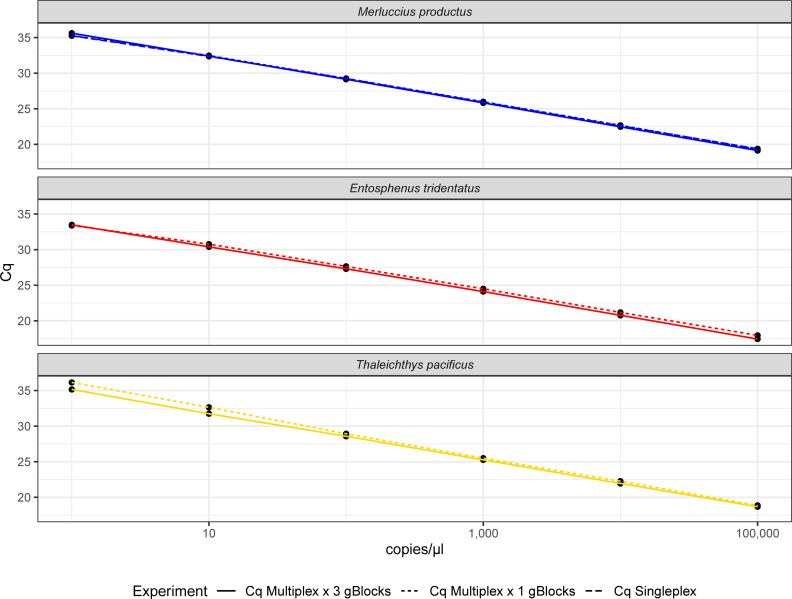
Comparison of the performance of the standards of the triple assay in a multiplex with 1 and 3 gBlocks per species. The performance for *Merluccius productus* in a singleplex is also represented.

Good efficiency of the qPCR (98.8–109%) and standard replication were observed since all qPCR run parameters fell within slope values of -3.35<m<-3.12 and r^2^ ≥0.99. The limit of detection (LOD) of all three assays at 95% or greater detection is below one copy μl^-1^ but could not be determined because no lower standards were tested, which corresponded to 10 fg μl^-1^ for the gDNA standards. Values of the C_q_ for the gBlocks followed the expected shift for ten-fold dilutions of 3.3 cycles (Fig 4). Eighty percent of the sampling negative controls from the validation subset had detectable signal of *M*. *productus* ranging from 0.2 to 2.2 copies μl^-1,^ which sets the limit of quantification (LOQ). Some lamprey signal at low levels (24 and 2 copies μl^-1^ on the 1 and 0.1 ng μl^-1^ standards, respectively) was found in the *T*. *pacificus* genomic DNA standards in agreement with the same sample used for the in vitro validation above.

Concentration values of *M*. *productus* found in all eDNA samples of this study (n = 50) ranged from 0 to 73.36 copies μl^-1^ and 44.64% were above the LOQ (n = 25) (S2 File). *T*. *pacificus* was also found in two samples out of the four biological replicates of the extraction test subset at <1 copy μl^-1^ from the site (120 m deep out of Cape Flattery) and absent in the validation subset. The finding of negligible amounts of *T*. *pacificus* among the extraction test subset is supported by the trawl survey conducted 30 h prior at this site, which documented 55.4% *M*. *productus*, 44.1% *G*. *chalcogrammus*, 0.2% *T*. *pacificus*, 0.2% *Oncorhynchus gorbuschaI* (Pink salmon), 0.1% *Oncorhynchus tshawytscha* (King Salmon), and 0.02% *Clupea pallasii* (Pacific herring) in a total of 935.3 kg. However, no formal quantitative analysis was attempted with these data because of the collection time differential.

**Fig 5:**
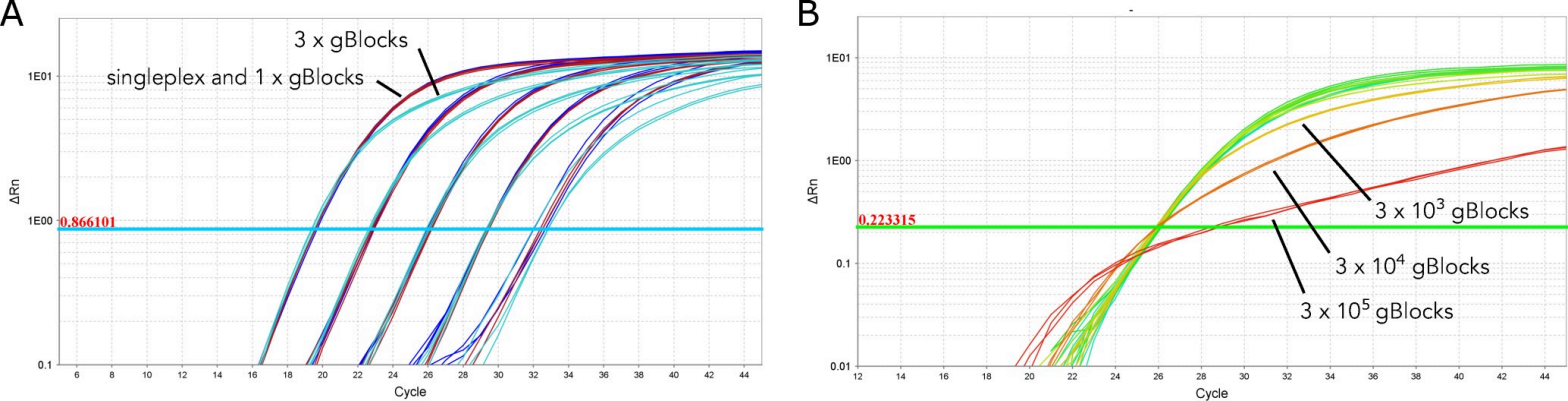
Amplification plot in logarithmic view. A. Comparison of the amplification of the Pacific hake gBlocks standards in a multiplex with the other two species combined and individually. B. Comparison of the amplification of the IPC for the non-template controls and the gBlocks standards combined at different concentrations (in copies μl^-1^) and some eDNA samples.

The amplification of the species-specific gBlocks standards individually and in the same well showed negligible C_q_ variation (Fig 4 and S2 File). Also, the addition of the other species primers and probes in the multiplex did not affect the amplification of the *M*. *productus* gBlocks when compared to *M*. *productus* gBlocks run individually and in a single reaction. Substantial difference in copy number was found for the genomic DNA standards (starting at 1 ng μl^-1^) depending on the species genome size (S2 File). The combination of the three gBlocks standards did not affect the reliability of the assay since it did not impact the exponential part of the amplification curve, nor the ΔRn of the plateau. However, it delayed the reach of the plateau phase most likely due to competition for the reagents in the master mix (Fig 5).

The IPC value for the highest gBlocks standards (100,000 copies), ran individually or combined, influenced the IPC amplification, most likely due to competition and exhaustion of reagents (Fig 5). The IPC amplification of the non-template controls was robust, with a standard deviation that ranged from 0.06 to 0.24 among PCR reactions (three to nine replicates, respectively). The threshold for the identification of inhibition (ΔC_q_ > ∂C_q ntc_) was thus set at 0.5. According to this parameter 17.85% of all eDNA samples (10 of 56 samples) showed inhibition with no apparent correlation with the chlorophyll a level (fluorescence values 0.05 to 8.98). The ΔCq of the IPC for the four most inhibited samples dropped from 17.19 to 0.71 after purification with inhibitor removal columns. None of the sampling (n = 10) and extraction negative controls (n = 3) showed signs of inhibition, hence the reagents in the PCI extraction method are ruled out as the source of inhibition. In addition, the vacuum grease used for the phase-lock is not a source of inhibitors since none of the samples of the phase-lock evaluation in S1 File showed signs of inhibition.

## Discussion

Environmental DNA monitoring holds promise as a non-invasive tool that can be included in the stock monitoring toolset [4]. The number of marine monitoring surveys using eDNA is rapidly increasing globally. There is a need for optimizing, validating and standardizing the methodology around eDNA for large-scale oceanic studies especially given the constraints of variable vessels with limited storage space and the desire for high sampling effort. DNA extraction protocols must maximize the recovery of good quality and high quantity DNA. The general trend for fish-targeted eDNA studies is to collect 1 or 2 L of surface water and capture eDNA on 0.7 μm GF filters, followed by extraction with the DNeasy Blood and Tissue Kit or PowerWater DNA Isolation Kit (Qiagen) of frozen filters, which has shown to yield high-quality eDNA and reliable results [31]. A larger volume of water (2.5 L) and a larger filter pore size (1 μm) were used here to maximize the DNA yield while avoiding filter clogging. The Longmire’s lysis buffer (see S3 File) is an inexpensive, safe and reliable lysis medium, favoring DNA preservation for prolonged periods of storage at ambient temperatures. It is also non-hazardous and convenient for transport. The 5 ml tube storage format for filters was chosen to facilitate the filter immersion in the buffer at sea with less manipulation by collectors and allows in-tube extraction of all suspended and filter bound materials. Commercial kits have their own proprietary lysis buffer to initiate the DNA extraction, but the samples used in this study were already stored in Longmire’s buffer. This required an adjustment of the original kit protocols (S1 File) that most likely affected the ideal performance of the DNA purification, since both the amount of DNA available for purification and the concentration of salts determining the binding capacity of the magnetics beads was likely not optimal for the performance of the commercial kit. Four commercial kits that exploit the capability of the nucleic acids binding a solid phase (either silica matrix or magnetic beads) in order to isolate the DNA, as well as the phenol:chloroform:isoamyl (PCI) separation method were evaluated in this study. The DNeasy Blood and Tissue kit (Qiagen) had been previously tested in our laboratory starting with filters stored in Longmire’s buffer with poorer results than the standard PCI (results not shown) and thus was disregarded in the search for a more cost-effective, reduced-labor method that yielded sufficient or more DNA. The best performing method in terms of total DNA and target DNA yield was the PCI method (16.13 and 5.17 × 10^−4^ ng μl^-1^ respectively), followed by the DNeasy PowerWater with frozen filters (5.67 and 9.02 × 10^−5^ ng μl^-1^) and GenFind v.3 (3.55 and 1.77 × 10^−4^ ng μl^-1^) (more in S2 File). The PCI method is the cheapest among all methods tested (as far as supply and reagents costs) and less than half the price of PW (details in S2 File). The integrity of the genomic DNA extracted with the PW kit was compromised, likely sheared due to the mechanical lysis procedure. Although DNA integrity could not be analyzed for MagAttract PowerWater DNA due to low yield, it is expected to be affected in the same way.

The PCI method is the most labor intensive and chemically hazardous of all methods tested. However, it is the most efficient method for the purposes of targeting specific species in the open ocean as described here. The PCI behaved less stochastically (showed the lowest C_q_ standard deviation) between biological and technical replicates, most likely due to the higher DNA concentration. As expected, the target DNA yield is a small fraction of the total DNA, hence the importance of enhancing the DNA concentration. The ratio of target DNA was not affected by the extraction method used indicating that the treatment does not affect the proportion of DNA of different taxa. The addition of the phase lock grease, although laborious, generates a higher yield and throughput because the aqueous layer containing the DNA can be quickly decanted into the next tube, reducing pipetting variation during recovery of the aqueous layer and exposure to fumes from the organic reagents. Mukhopadhyay and Roth [32] recovered 20% more total DNA with their phase-lock protocol; however, the improvement could be very variable between samples, replicates, number of steps in the DNA purification method, DNA concentration and the expertise of the person performing the protocol.

The exogenous internal control provides confidence on the identification of false negative or reduced quantification caused by inhibition and enables identification of samples that need further treatment. Equal levels of inhibition were observed in biological replicates showing consistency in the inhibition co-purification. Inhibition was observed in 18% of the eDNA samples. The origin was not ascertained here. However, this issue was circumvented by purifying the DNA with inhibitor removal columns with an additional cost per sample (S1 File). The shift of ΔC_q_ ≥ 3 is often applied in the eDNA field [33–35] as the cut-off to account for inhibition. That level is disregarded here and anticipated to skew the quantification results with almost a log-loss in copy number. The use of an inhibitor-tolerant master mix should be favored to avoid bias caused by purification or dilution treatments [36].

The use of multiple synthetic oligonucleotides (gBlocks) in the same well as standards of the qPCR enabled accurate quantification of target copy number instead of an inference from genomic DNA standards. It also reduced considerably the number of pipetting steps and standard preparation time. Synthetic oligonucleotides are a good substitute for genetic laboratories with no plasmid cloning capabilities. *Merluccius productus* contamination at very low levels was found in the sampling negative controls. Although sparse replication and low amplification was observed, it signals underlying contamination at collection. This is unsurprising given the operations of the survey typically target this species and daily trawls brought large amounts on board. Stricter contamination measures, further tests and improvements should be pondered in future sampling campaigns. Interspecific contamination was also found in the reference samples used to test the specificity of the triplex assay. This was most likely due to insufficiently clean collection measures at fishing trawl campaigns in which the specimens are handled and subsampled swiftly. These findings show the sensitivity of the assays and call for future reference material to be decontaminated prior to the DNA extraction by submerging the samples in bleach [37]. Despite the above-mentioned findings, the triple assay was found to be specific, confirmed by the fact that the extraction test subset of samples was taken at a site where a mixed school of *M*. *productus* and *G*. *chalcogrammus* was observed. Moreover, negligible levels of *T*. *pacificus* were found and no lamprey was detected for the extraction test subset as expected from the trawl observations. *M*. *productus* was present in 44% of the survey eDNA samples analyzed (validation subset samples) while the other two species were absent in agreement with the total trawl catch in de Blois [38].

In summary, we describe an approach for the quantification of multiple species of fish in oceanic water that can be used in large fishery surveys. In addition to the logistics for collection and filtration used, the use of synthetic oligonucleotides as qPCR standards in a multiplex in combination with an exogenous internal positive amplification control made this assay practical, quantitative, sensitive and reliable. This represents a validated method for the isolation and analysis of environmental DNA (Fig 1 and S3 File) with promising prospects for fish monitoring.

## Supporting information

S1 File Supplemental methods and results. Methods results, primers and gBlocks sequences, costs and reference material used in this study.
(PDF)

S2 File Primer details and results. (XLSX)

S3 File Complete workflow and protocols. Complete collection, DNA extraction and quantification protocols.
(PDF)
