# Supplementary material for: A workflow for the relative quantification of multiple fish species from oceanic water samples using environmental DNA (eDNA) to support large-scale fishery surveys
Source: PLoS One. 2021 Sep 27;16(9):e0257773. doi: 10.1371/journal.pone.0257773 (PMC8476043; doi:10.1371/journal.pone.0257773)
Supplement: S1 File — Methods results, primers and gBlocks sequences, costs and reference material used in this study. (PDF) [file pone.0257773.s001.pdf]

A workflow for the relative quantification of multiple fish species from oceanic water samples using environmental DNA (eDNA) to support large-scale fishery surveys

Ana Ramón-Laca, Abigail Wells, Linda Park

Supporting material S1 – Supplemental methods and results, DNA extraction methods tested, primers and gBlocks sequences, costs and reference material used in this study

1. Supplemental methods and results

2. Extraction methods used with the modifications implemented in this particular study:
   2.1. Phenol-chloroform-isoamyl alcohol DNA purification
   2.2. Qiagen DNeasy PowerWater kit
   2.3. Qiagen MagAttract PowerWater kit
   2.4. Agencourt DNAdvance (Beckman Coulter)
   2.5. Agencourt GenFind v.3 (Beckman Coulter)

3. DNA extraction methods cost breakdown

4. Final DNA extraction method: Phenol-chloroform-isoamyl alcohol DNA purification

5. Genomic DNA electrophoresis visualization and target DNA quantification comparison between methods

6. gBlocks sequences and primers

7. List of species subjected to in silico and in vitro specificity tests

8. Specificity rates from the in vitro specificity tests

9. Specificity tests ran with primerTree

1. Supplemental methods and results

Methods

To develop a species-specific detection and quantification assay, sequences from four mitochondrial genes for each species were downloaded from GenBank: 12S rRNA (12S), 16S rRNA (16S), cytochrome oxidase subunit 1 (COI) and cytochrome b (cytb). Sequences examined included multiple individuals (when available) of the three species of interest – Pacific hake (*M. productus*), Pacific lamprey (*E. tridentatus*) and eulachon (*T. pacificus*)– as well as of closely related species expected to occur sympatrically (Table S1.6). All sequences were aligned using the analysis software Geneious Prime (www.geneious.com). Highly dissimilar regions for species discrimination were identified by eye and sets of primers and probes were designed aiming for a 40-60 % GC content in primers and probes, a short amplicon (≤100 base pairs) and a fragment melting temperature (T_m) of ca. 85 °C. Primers and hydrolysis probes were checked for hairpin, self-dimer (T_m < 50°C) and hetero-dimer formation using the built-in primer tool in Geneious Prime and PrimerDimer (Lu *et al.* 2017).

To first assess potential amplification inhibition and to test the qPCR chemistry, a fourth PCR replicate of the DNA from the extraction test subset of samples isolated via the PCI method was run in which 2 µl of the standard at 1 ng µl^{-1} was spiked as an internal inhibition control. Inhibition occurrence was measured as a shift in C_q, ΔC_q of a given sample was calculated as the difference between the C_q value of the replicate of the sample that had been spiked with the standard and the average of the C_q of the three replicates of the 1 ng µl^{-1} standard. A sample was determined inhibited if it had a positive ΔC_q value, while a non-inhibited sample showed a negative value. Two different master mixes were compared: the TaqMan® Environmental Master Mix 2.0 and the TaqPath ProAmp Multiplex Master Mix (ThermoFisher Scientific, Waltham, MA, U.S.). The latter is a more affordable master mix, intended to outperform in the presence of inhibitors, using a passive reference in the deep red channel (Mustang Purple dye), allowing for another dye/target in the ROX channel thus expanding the multiplexing capabilities.

To increase the throughput of the PCI extraction and decrease repetitive pipetting for the anticipated 1800+ samples collected, the use of silicone vacuum grease as a phase-lock (Mukhopadhyay & Roth 1993) between the organic and aqueous phases was evaluated. For three samples the lysis buffer and the filters within these samples were split into two equal volume subsamples: one subsample was treated with the addition of ca. 500 µl of high-vacuum silicone grease (Dow Corning, Midland, MI, US), creating an interphase layer allowing easy decanting of the aqueous layer (upper phase) into the next extraction step (de Blois 2020), for the other sample the aqueous phase was pipetted manually, avoiding the organic interphase by eye. DNA from both treatments was eluted in a final volume of 50 ml of TlowE. In addition, a mock mixture of DNA using the gBlocks of the three target species genes at 1000 and 10,000 copies in 2 ml of Longmire buffer was set up to assess the difference in DNA recovery with the phase lock. Eight samples at each concentration were extracted per treatment (i.e., with and without the phase lock) and then evaluated with the multiplex assay on the qPCR.

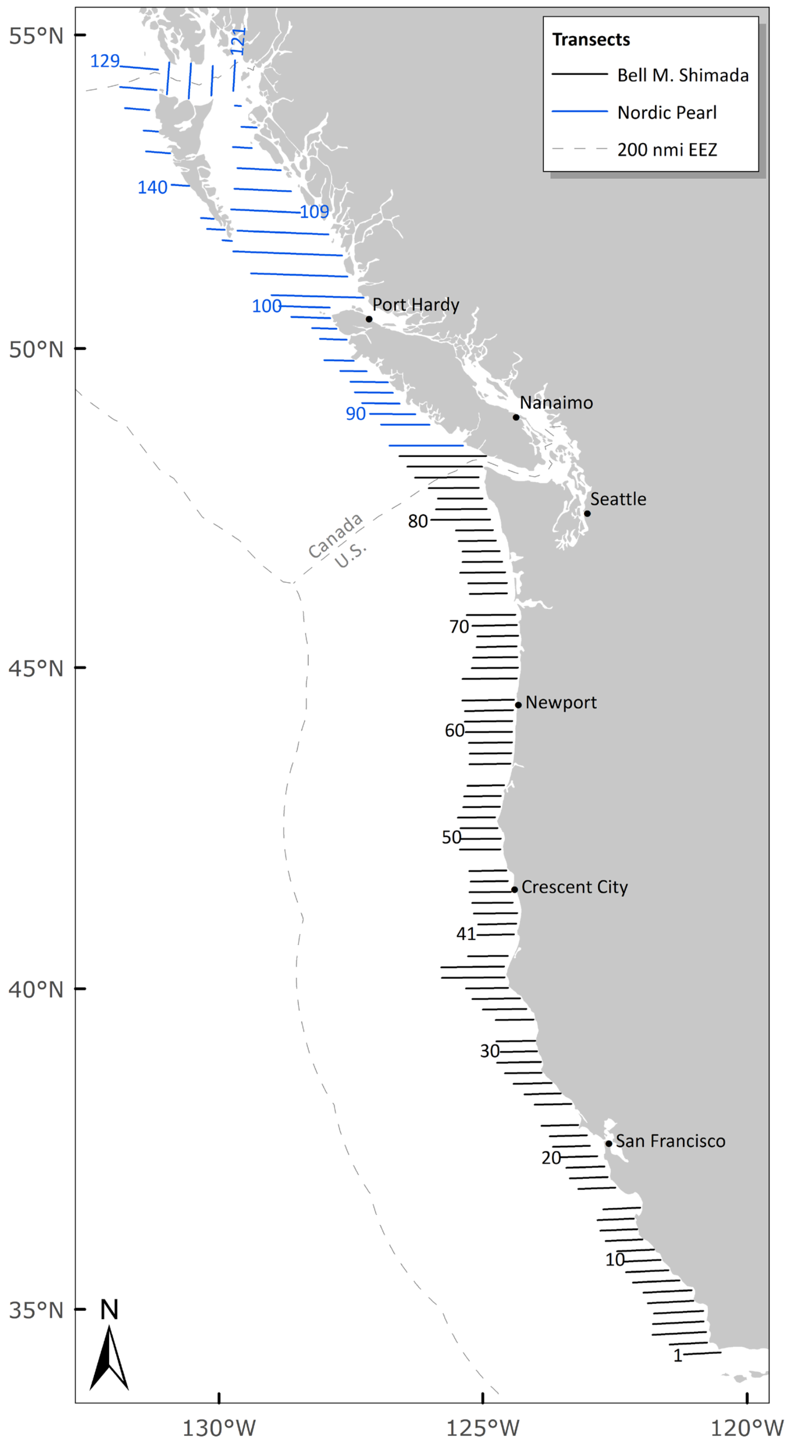

Figure S1. Survey track design used during the 2019 Joint U.S.–Canada Integrated Ecosystem and Pacific Hake Acoustic-Trawl Survey. eDNA samples were scattered throughout transects 22 to 85. [From NOAA Processed Report NMFS-NWFSC-PR-2020-03 (de Blois 2020)]

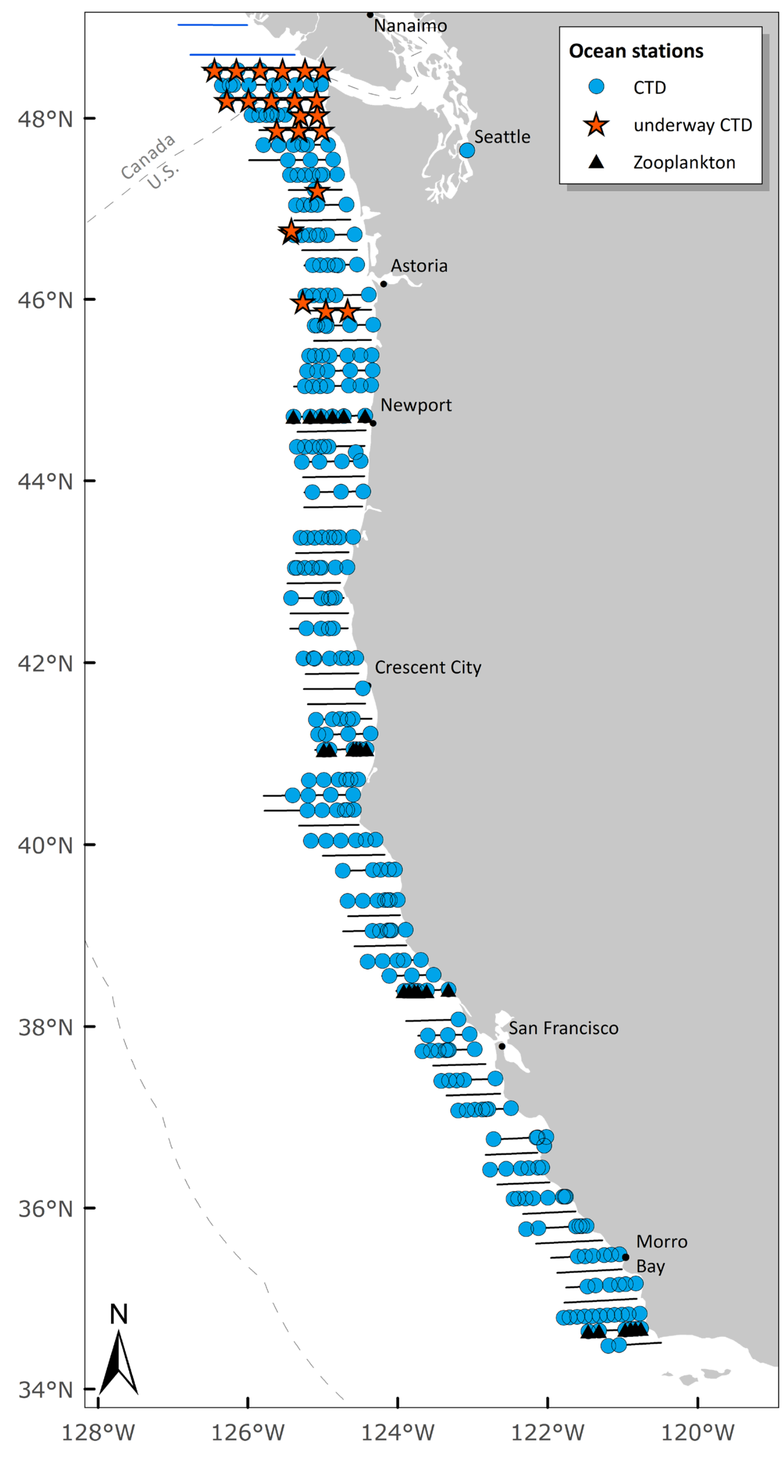

Figure S2. Acoustic transect lines with locations of zooplankton stations, conductivity-temperature-depth (CTD) rosette deployments, and underway CTDs (uCTDs) conducted by the RV *Shimada* during the 2019 Joint U.S.–Canada Integrated Ecosystem and Pacific Hake Acoustic-Trawl Survey. [From NOAA Processed Report NMFS-NWFSC-PR-2020-03 (de Blois 2020)]

Results

Sequence variation at cytb and 16S was not optimal in these species for primer design and assays for these regions were not developed. Details of the primers and probes used in the final assay are found below.
The TaqPath ProAmp Multiplex Master Mix outperformed the Environmental TaqMan master mix in terms of inhibition occurrence with a drop in ΔC_q from 0.73 (±0.43) to -0.10 (±0.15) for the subset of samples extracted with the PCI method.

The addition of the phase lock to the PCI extraction method yielded more target DNA for all species at both concentrations tested in the known-copy-number experiment. However, the gain is variable depending on the starting concentration (results in S2). The DNA recovered was much lower than the concentration added, which is hypothesised to be due to hindered precipitation for short fragments of DNA at low quantities without carrier DNA as in Hunter *et al.* (2019). A gain of ca. 100 % in the higher concentration was obtained for each species with the phase-lock (124.99 % for Pacific hake, 117.7% for Pacific lamprey and 98.55% for eulachon) while the gain for the lower concentration was more variable (4.78 % for Pacific hake, 13.69% for Pacific lamprey and 63.71% for eulachon. The addition of the silicone vacuum grease did not affect the amplification of the IPC and thus it is not a source of inhibition in itself.

2. Extraction methods used with the modifications implemented in this particular study

2.1 Phenol-chloroform-isoamyl alcohol DNA purification

Step		Details
1	**collection**	2.5 l filtered immediately after collection using a 1µm MCE filter, 47 mm ∅
2	**preservation**	5 ml tube - 2 ml of Longmire lysis buffer at room temp
3	**heat-shock**	95°C for 5' and then allow samples to go to room temperature
4	**digestion**	proteinase K (final concentration 2mg/ml) (i.e., ~200 ul in 2ml of Longmire)
5	**incubation**	56°C at 120 rpm for 2h
6	**PCI (25:24:1)**	add 2 ml of phenol-chloroform-isoamyl (25:24:1) with 10mM Tris, pH 8.0, 1mM EDTA
7	**centrifugation**	shake and spin 13.3 x g for 5' at 4°C
8	**CI**	aqueous layer into new tube and add 2.2 ml of chloroform:isoamyl (24:1)
9	**centrifugation**	shake and spin 13.3 x g for 5' at 4°C
10	**CI**	aqueous layer into new tube and add 2.2 ml of chloroform:isoamyl (24:1)
11	**centrifugation**	shake and spin 13.3 x g for 5' at 4°C
12	**Isopropanol**	aqueous layer into new tube and 2 ml of isopropanol and 80 µl of 5M NaCl
13	**mixing**	invert several times
14	**precipitation**	overnight at -20°C
15	**centrifugation**	spin 13.3 x g for 30' at 4°C
17	**wash**	pour liquid off slowly and add 800 µl of ice cold 70% EtOH

18	**centrifugation**	shake and spin 13.3 x g for 5' at 4°C
19	**drying**	pour liquid off slowly and let tubes to dry for 1 h
20	**resuspension**	once they are dry, resuspend in 100 µl TE buffer (warm - 37°C)
21	**storage**	and store in the freezer (-20 °C)

2.2 Qiagen DNeasy PowerWater kit (Cat. No. 14900-50-NF)

Step		Details
1	**collection**	2.5 l filtered immediately after collection using a 1µm MCE filter, 47 mm ∅
2	**preservation**	Filter in a 5 ml tube at -80°C
3	**filter storage**	transfer the beads in the PW DNA bead tube to the tube with the filter
4	**lysis**	add 1 ml of PW1 (at 55°C) - vortex for 5' max speed
5	**incubation**	65°C for 10' to aid lysis of algae or fungi
6	**optional step**	centrifuge 4000 x g for 1'
7	**transfer**	supernatant to a clean 2ml tube (expect to recover 600-650ul)
8	**centrifugation**	centrifuge 13,000g for 1', important!
9	**transfer**	supernatant to a clean 2ml tube (avoiding pellet)
10	**inhibitor removal**	add 200 µl of IRS and incubate at 5°C for 5'
11	**centrifugation**	centrifuge 13,000g for 1'
12	**transfer**	supernatant to a clean 2ml tube (avoiding pellet)
13	**addition**	650 µl of PW3 and vortex briefly
14	**binding**	load the 650 µl in a column and centrifuge at 13000 x g for 1'
15	**wash**	new collection tube and add 650 µl of PW4 and centrifuge at 13000 x g for 1'
16	**wash**	new collection tube and add 650 µl of ethanol and centrifuge at 13000 x g for 1'
17	**ethanol removal**	discard flow-through and centrifuge at 13000 x g for 2'
18	**elution**	transfer column to a final tube, add 100 µl EB and centrifuge at 13000 x g for 1'
19	**storage**	store in the freezer since EB (does not contain EDTA, only 10mM Tris)

2.3 Qiagen MagAttract PowerWater kit (Cat. No.27800-4-EP)

Step		Details
1	**collection**	2.5 l filtered immediately after collection using a 1µm MCE filter, 47 mm ∅
2	**preservation**	5 ml tube - 2 ml of Longmire lysis buffer at room temp
3	**beads addition**	split the volume in two 2 ml tubes (one with the filter), add beads
4	**lysis**	bead beater for 5' at 20Hz, rotate cassettes and shake again
5	**centrifugation**	centrifuge 4500 x g for 1', combine supernatant to a clean tube

6	**inhibitor removal**	add 200 µl of IRS solution, vortex and incubate at 5°C for 5'
7	**centrifugation**	centrifuge 4500 x g for 6', avoiding pellet supernatant to a clean tube
8	**centrifugation**	centrifuge 4500 x g for 6', avoiding pellet up to 850 µl supernatant to a clean tube
9	**optional digestion**	30 µl of proteinase K and incubate at 65°C for 10'
10	**stop point**	if necessary, keep the plate in the fridge overnight
11	**binding**	dispense and mix (10 times) 470 µl per sample of ClearMag Bead-Bind mix
12	**separation**	mix for 10', magnet for 5' and aspirate 900 µl and discard
13	**wash**	500 µl of ClearMag wash and mix (5-10 times) until resuspended
14	**separation**	mix for 5', magnet for 5' and aspirate supernatant and discard
15	**wash**	repeat steps 13-14
16	**wash**	300 µl of ClearMag wash and mix, magnet and aspirate supernatant
17	**elution**	once they are dry, resuspend in 100 µl of TE, mix 5', magnet for 3' and keep supernatant
18	**storage**	and store in the freezer (-20 °C)

2.4 Agencourt DNAdvance (Beckman Coulter) - Cat. No. A48705

Step		**Details**
1	**collection**	2.5 l filtered immediately after collection using a 1µm MCE filter, 47 mm ∅
2	**preservation**	5 ml tube - 2 ml of Longmire lysis buffer at room temp
3	**heat-shock**	95°C for 5' and then allow samples to go to room temperature
4	**proteinase k prep**	3.25 ml of proK buffer to proK bottle once at first
5	**digestion**	add 100 µl of proK and incubate at 37°C at 120 rpm for 2 h
6	**transfer**	split lysate into 2 × 2 ml tubes
7	**addition of beads**	add 500 µl of beads solution, mix well to each (up to 15 times avoiding bubbles)
8	**incubation**	at room temperature for 1'
9	**isolation**	magnet for 4' and discard supernatant
10	**washing × 2**	resuspend (up to 20 pipetting steps) 2 × with 340 µl of 70% ethanol and then allow to dry for 5' out of the magnet
11	**elution**	once they are dry, resuspend in 55 µl EBA buffer (warm - 37°C) out of the magnet and mix well, magnet for 5' and take 50 µl from each
12	**storage**	and store in the freezer (-20 °C)

2.5 Agencourt GenFind v.3 (Beckman Coulter) – Cat. No. C34881

Step		**Details**
1	**collection**	2.5 l filtered immediately after collection using a 1µm MCE filter, 47 mm ∅
2	**preservation**	5 ml tube - 2 ml of Longmire lysis buffer at room temp

3	**heat-shock**	95°C for 5' and then allow samples to go to room temperature
4	**heat-shock**	95C for 5' and then allow samples to go to room temperature
5	**digestion**	add 100 µl of proteinase K and incubate at 37°C at 120 rpm for 2 h
6	**incubation**	37C at 120 rpm for 2 h
7	**transfer**	split lysate into 2 × 2 ml tubes
8	**DNA binding**	add 300ul of beads **to each and** mix well (up to 10 times avoiding the formation of bubbles)
9	**incubation**	at room temperature for 5'
10	**isolation**	magnet for 20' and discard supernatant
11	**washing × 2**	off the magnet resuspend in 800 µl of WBB by mixing, magnet for 10'
12	**washing × 2**	off the magnet resuspend in 800 µl of WBC by mixing (prepared first time by adding ethanol), magnet for 8'
13	**elution**	once they are dry, resuspend and combine in 110 µl EB (warm - 37C) off the magnet, mix well, incubate for 2'. Magnet for 2' and take 100 µl
14	**storage**	and store in the freezer (-20 °C)

3 DNA extraction methods cost breakdown per sample

Phenol:Chloroform:Isoamyl	quantity		$ cost	Total $
proteinase k (20 mg μl^{-1})	100.00	µl	1.43	**4.29**
Silicone vacuum grease	3	ml	0.50	
P:C:I (25:24:1)	2.00	ml	0.97	
C:I (24:1)	4.50	ml	0.38	
Isopropyl	2.00	ml	0.06	
ethanol	1.12	ml	0.02	
5 ml LoBind tubes Eppendorf	3.00		0.89	
**Powerwater (Qiagen)**	**quantity**		**$ cost**	**Total**
PowerWater kit -Qiagen			10.24	**10.39**
1.5 ml low bind tubes Eppendorf	1.00		0.15	
**MagAttract Powerwater (Qiagen)**	**quantity**		**$ cost**	**Total**
MagAttract PowerWater Qiagen	1.00		5.03	**5.19**
protainase k	30.00	µl		
1.5 ml low bind tubes Eppendorf	1.00		0.15	
**DNAdvance (Agencourt)**	**quantity**		**$ cost**	**Total**
Agencourt DNAdvance	1.00		12.50	**12.65**
1.5 ml low bind tubes Eppendorf	1.00		0.15	
**GenFInd (Agencourt)**	**quantity**		**$ cost**	**Total**
Agencourt GenFind v.3	1.00		6.79	**6.94**
1.5 ml low bind tubes Eppendorf	1.00		0.15	
One-step inhibitor removal (Zymo)	**quantity**		**$ cost**	**Total**
Columns	1.00		2.16	**2.16**
Plates (cost per sample)	1.00		1.69	1.69

4 Final DNA extraction method: *Phenol-chloroform-isoamyl alcohol DNA purification*

Step		Details
1	**collection**	2.5 l filtered immediately after collection using a 1µm MCE filter, 47 mm ∅
2	**preservation**	2 ml of Longmire lysis buffer in a 5 ml LoBInd Eppendorf tube stored at ambient temp
3	**heat-shock**	95°C for 5' and then allow samples to cool to room temperature
4	**digestion**	add 100 µl proteinase K (final concentration 1 mg/ml)
5	**incubation**	56°C at 120 rpm for 2h
6	**phase lock set up**	add ~800 µl of vacuum grease with a syringe onto the wall of the tube of the 5 ml tube
7	**PCI (25:24:1)**	add 2 ml of phenol-chloroform-isoamyl (25:24:1) pH8
8	**centrifugation**	shake well and spin 13.3 x g for 5' at 4°C
9	**phase lock set up**	place ~800 µl of vacuum grease in 2 sets of empty tubes for CI. (These can be prefilled for convenience)
10	**CI**	add 2.2 ml of chloroform:isoamyl 24:1 and decant aqueous layer from step 6. **Important**: only add the chloroform just before use (while the tubes with PCI are in the centrifuge) so it does not affect the phase lock if grease is at the bottom.
11	**centrifugation**	shake and spin 13.3 x g for 5' at 4°C
12	**CI**	add 2.2 ml of chloroform:isoamyl 24:1 and decant aqueous layer from step 9
13	**centrifugation**	shake and spin 13.3 x g for 5' at 4°C
14	**Isopropanol**	add 2 ml of isopropanol (can be prefilled for convenience), add 80 µl of 5M NaCl and decant aqueous layer from step 11
15	**mixing**	invert several times
17	**precipitation**	overnight (or 2 h) at room temperature
18	**centrifugation**	spin 13.3 x g for 30' at 4°C
19	**wash × 2**	pour liquid off slowly and add 800 µl of ice cold 70% EtOH
20	**centrifugation**	shake and spin 13.3 x g for 5' at 4°C **and repeat wash**
21	**drying**	pour liquid off slowly and allow tubes to dry for 1 h or until dry
22	**resuspension**	once they are dry, resuspend in 100 µl TE buffer (warm - 37°C)
23	**storage**	store in the freezer (-20-80 °C)

5 Genomic DNA electrophoresis visualization and target DNA quantification comparison between methods

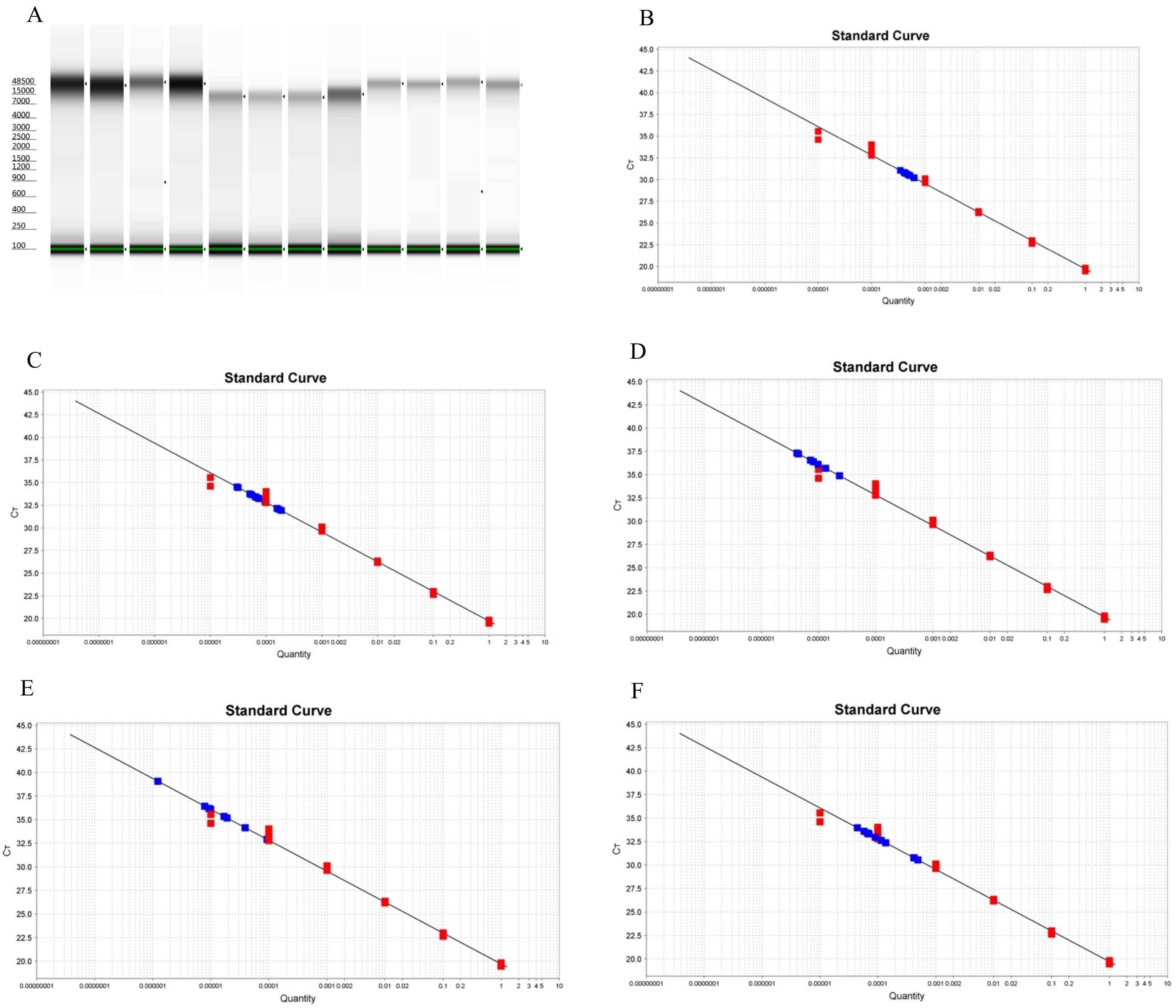

Results from the DNA extraction methods comparison (qPCR 1): A. TapeSTattion results for samples extracted with PCI (lanes 1-4), DNeasy PowerWater (lanes 5-8) and Agencourt GenFind v.3 (lanes 9-12), samples from the other two methods were barely visible and thus are not shown here; figures B to E: quantification of target DNA (i.e., hake) on the qPCR with the experiment samples in blue and the standards in red [slope = -3.275, r^2 = 0.99, efficiency = 102 %, y-intercept = 20.053] B. PCI; C. DNeasy PowerWater; D. MagAttract PowerWater, E. Agencourt DNAdvance; F. Agencourt GenFind v.3

6 gBlocks sequences and primers

>Et-COI-230F
TGATATAGCCTTCCCTCGTATAAACAAC
>Et-COI-296P
AAGTAGGAGTAGTGAGGGTGGAAGTAA
>Et-COI-323R
CCCTGCTTCAACTCCTGC
>Moa-IPC-F
CCATCAGACCATACAGTGCAA
>Moa-IPC-P
CCGAGATTGTGGGCGCCTGGGTTAC
>Moa-IPC-R
GCACTTCAGGTGTATAACTAATGAACG
>Mp-12S-59F
AAATGTTTAAACTAGAGCCGAATAGC
>Mp-12S-102P
CACTCGAGGCCACGAAGTACAATT
>Mp-12S-158R
TCGTGGAGTCAAAGTGGGGTAGA
>Tp-COI-F
CCTTATTCGTCTGAGCCGTCCTG
>Tp-COI-P2
GGCCGTTCTTCTCCTCCTTTCCCTCCCAGTTT
>Tp-COI-R
GTTAAGATTTCGGTCTGTTAGAAGCATA
>Euryapteryx_curtus_D-loop_IPC_gBlocks
GGCTCATGCCCATCAGACCATACAGTGCAATCGGACACGTTTCCCTTGCTCCCCTCTCGTAA
CCCAGGCGCCCACAATCTCGGGGTGCTTGAAAGCCATACGTTCATTAGTTATACACCTGAA
GTGCACA
>Entosphenus_tridentatus_COI_gBlocks
TCATACTTAGTGCCCCTGATATAGCCTTCCCTCGTATAAACAACATAAGCTTTTGATTACTTC
CACCCTCACTACTCCTACTTTTAGCCTCCGCAGGAGTTGAAGCAGGGGCTGGCACAGGATG
AACCGT
>Merluccius_productus_12S_gBlocks
AAGCGTGGTTAAAAAAATGTTTAAACTAGAGCCGAATAGCCTCAAAGCAGTTATACGCACTC
GAGGCCACGAAGTACAATTACAAAAGTAGCTCTACCCCACTTTGACTCCACGAAAACCATAA
AACAAA
>Thaleichthys_pacificus_COI_gBlocks
AGTACCAGACCCCCTTATTCGTCTGAGCCGTCCTGATTACGGCCGTTCTTCTCCTCCTTTCC
CTCCCAGTTTTAGCTGCTGGAATTACTATGCTTCTAACAGACCGAAATCTTAACACCACTTTC
TTTGA

7 **List of species subjected to in silico and in vitro specificity tests**. Target species are highlighted in bold. N: number of samples used for the in vitro test. GenBank Accession numbers are are listed for the sequences used for primer design and in silico specificity tests for COI, 12SrRNA and D-loop. Individuals tested in vitro do not coincide with sequences tested in silico. *Not subjected to in vitro tests.

			Accession number		
Scientific name	Common name	N	COI	12SrRNA	D-loop
Entosphenus tridentatus	**Pacific lamprey**	33	**HQ579095-HQ579096, HQ579132, JN025332, KF918874-KF918875, KF929845, KX389871- KX389875, KY570333**	LCO91545-LCO91546	
Lampetra ayresii	Wester river lamprey	1	This study	LCO91547	
Entosphenus similis	Klamath lamprey		JN025329-JN025331		
Merluccius productus	**Pacific hake**	39	EU489713, FJ164843- FJ164858, GU440405, JQ354224- JQ354225, KF918882, KM019356- KM019362, KX017717- KX017719, KX017722-KX017723, KX017727	**DQ274012-DQ274013, DQ533243, LC069504, LC091814, LC091816-LC091817**	
*Merluccius gayi**	Peruvian hake	1		FJ215054	
Albatrossia pectoralis			JQ353957-JQ353960, KX656364-KX656366	FJ214987, LC049856	
Antimora microlepis			FJ164298-FJ164302, JF952669, KF918862-KF918863, KF929605	AB018228, LC049860	
Coryphaenoides acrolepis			FJ164488-FJ164496, JQ354060-JQ354061, KF420483	FJ214988, AB018228, LC049860	
Boreogadus saida	Polar cod	2			
Gadus macrocephalus	Pacific cod	4	FJ164608-FJ164619, GU324188, HQ712374- HQ712383, JQ354097-JQ354101, KF929903-KF929904, KF965345-KF965349, KF965384-KF965388	FJ215032, FJ620138, KP644362-KP644363, LC021203, LC091826-LC091827	
Gadus chalcogrammus	Alaska pollock	3	AB094061, AB182300-AB182308	FJ215085, KP644356-KP644357, LC021202, LC091821-LC091823	
Microgadus proximus	Pacific tomcod	4	FJ164859- FJ164865, GU440406, JQ354226- JQ354229	FJ215062, LC091824-LC091825	
Thaleichthys pacificus	**Eulachon**	45	**JQ354514- JQ354516, KF918978**	AY430262, EU621501, KM273861, KM282450, LC091603, LC091616	
Spirinchus thaleichthys	Longfin smelt		KF196155-KF196157	AY430259, EU621499-EU621500, LC091612-LC091615	
Spirinchus starksi	Night smelt		EF609475, GU440528-GU440529, JQ354501, KF196151-KF196154	EU621498, LC091604, LC091610-LC091611	
Allosmerus elongatus	Whitebait smelt		FJ164251-FJ64263, JQ353963-JQ353964	EU621477-EU621478, LC091607-LC091608	
Hypomesus olidus	Pond smelt		HQ712490		
Hypomesus pretiosus	Surf smelt		JQ354135-JQ354138	EU621485-EU621486	
Mallotus villosus	Capelin		JQ354218	EU621489-EU621490	
Osmerus mordax	Rainbow smelt		JQ354253	EU621491-EU621492	
Euryapteryx curtus	**Broad-billed moa**				GU139002

8 Specificity rates from the in vitro specificity tests

test	species	TPR	FPR	TNR	FNR
initial	Eulachon	1	0	1	0
initial	Pacific hake	1	0.054	0.946	0
initial	Pacific lamprey	1	0.051	0.950	0
final	Eulachon	1	0	1	0
final	Pacific hake	1	0	1	0
final	Pacific lamprey	1	0	1	0

TPR: True positive rate; FPR: False positive rate; TNR: True negative rate; FNR: False negative rate

9 Specificity tests ran with primerTree

```
library(primerTree)
library(tidyverse)
library(kableExtra)
```

Primer pairs specificity test using primerTree

The following commands are the steps used to look at the specificity of each pair of primers using the R package primerTree by Cannon *et al.* (2016). The package tests a pair of primers in silico against the NCBI database.

The default settings allow 3 mismatches in each primer to six mismatches between the two primers. The script has been adapted here to be able to control the number of mismatches.

Here one or fewer mismatches per primer have been allowed.

Primers pairs are:

Tested in pairs of forward and reverse per species. Probes are not tested here.

```
EtCOI230F <- "TGATATAGCCTTCCCTCGTATAAACAAC"
EtCOI323R <- "CCCTGCTTCAACTCCTGC"
MpIPCF <- "CCATCAGACCATACAGTGCAA"
MpIPCR <- "GCACTTCAGGTGTATAACTAATGAACG"
Mp12S59F <- "AAATGTTTAAACTAGAGCCGAATAGC"
Mp12S158R <- "TCGTGGAGTCAAAGTGGGGTAGA"
TpCOIF <- "CCTTATTCGTCTGAGCCGTCCTG"
TpCOIR <- "GTTAAGATTTCGGTCTGTTAGAAGCATA"
```

To build a table with the taxonomy by genus and with Acc #

```
## the default table only gives the genBank identifier (gi), not the accession # (access
ion). An intermediate table has to be generated to blend the information from BLAST resu
lt and taxonomy outputs from Primer.pair

Recover_taxonomy_list <- function(Primer.pair) {
```

```
  Primer.pair$taxonomy %>%
  left_join(Primer.pair$BLAST_result,by="gi")  %>%
  distinct(order, genus, species, accession) %>%
  arrange(desc(genus))}
```

Pacific lamprey

```
Primer.pair.Et <- search_primer_pair(
EtCOI230F,
EtCOI323R,
name = NULL,
num_aligns = 500,
  num_permutations = 25,
  simplify = TRUE,
  clustal_options = list(exec = "clustalo", quiet = TRUE, original.ordering = TRUE),
  distance_options = list(model = "N", pairwise.deletion = T),
  api_key = "api.key.number",
  .parallel = FALSE,
  .progress = "none")
write_rds(Primer.pair.Et, "Et_search.rds")
Primer.pair.Et <- read_rds("Et_search.rds")
```

Filter for 1 or 0 mismatches for each primer and recover the list of results

```
Primer.pair.Et$BLAST_result %>%
 filter (mismatch_forward <= 1 & mismatch_reverse <= 1) %>% pull(gi) -> Primer.pair.Et.f
iltered


Primer.pair.Et->Et.seqs
Primer.pair.Et$sequence[Primer.pair.Et.filtered] -> Et.seqs$sequence
clustalo(Et.seqs$sequence)->Et.seqs$alignment
tree_from_alignment(Et.seqs$alignment)->Et.seqs$tree
Et.seqs$taxonomy <- get_taxonomy(Primer.pair.Et.filtered)


Recover_taxonomy_list(Et.seqs) -> Et.NCBI.PCR.list


Et.NCBI.PCR.list %>%
  write_csv("Et.NCBI.PCR.list.csv")


Et.NCBI.PCR.list %>%
  group_by(species) %>%
  count()
```

```
## # A tibble: 6 x 2
## # Groups:   species [6]
##   species                      n
##   <chr>                    <int>
## 1 Entosphenus lethophagus      3
## 2 Entosphenus similis          2
## 3 Entosphenus tridentatus     13
## 4 Lampetra ayresii             1
## 5 Phyllobates aurotaenia       1
## 6 Trichogaster fasciata        1
```

Plot tree

```
plot_tree (Et.seqs$tree, main= "Pacific lamprey in silico PCR", rank="species", taxonomy
= Et.seqs$taxonomy, guide_size = 10, legend_cutoff = 50) +
 theme_minimal() +
  labs(x = "", y = "")+
  ggsave("Et.insilicoPCR.NCBI.jpeg")
```

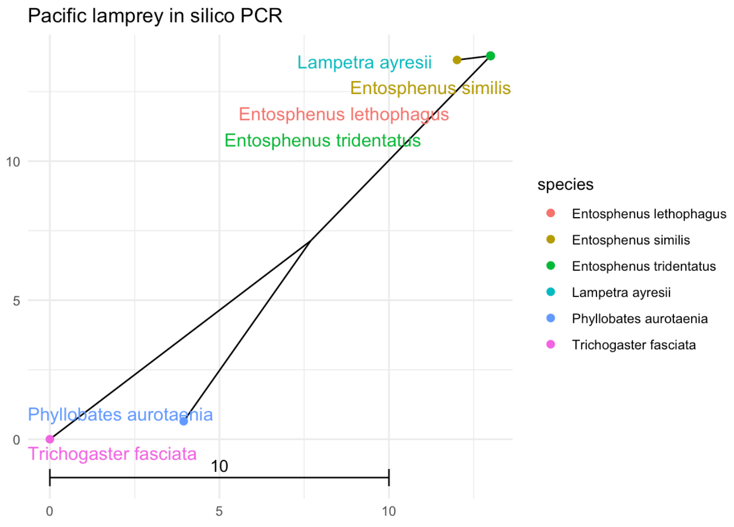

Broad-billed Moa

```
## # A tibble: 2 x 2
## # Groups:   species [2]
##   species                 n
##   <chr>               <int>
## 1 Euryapteryx curtus     58
## 2 Euryapteryx gravis      1
```

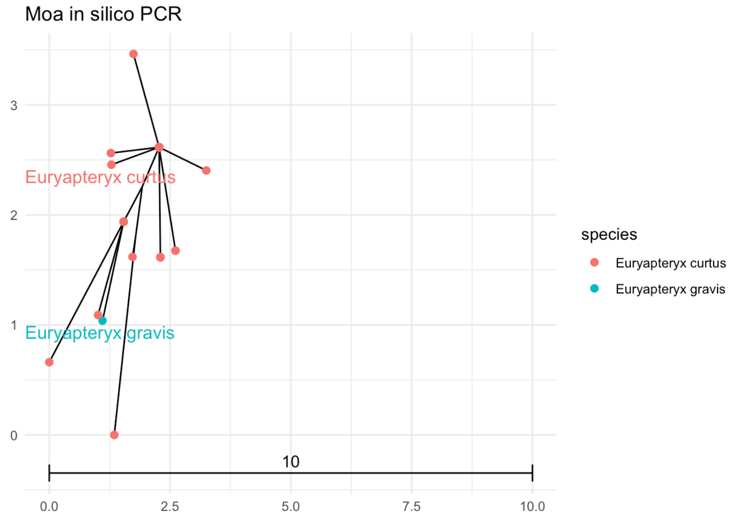

Pacific hake

```
## # A tibble: 2 x 2
## # Groups:   species [2]
##   species                   n
##   <chr>                 <int>
## 1 Merluccius gayi           1
## 2 Merluccius productus      6
```

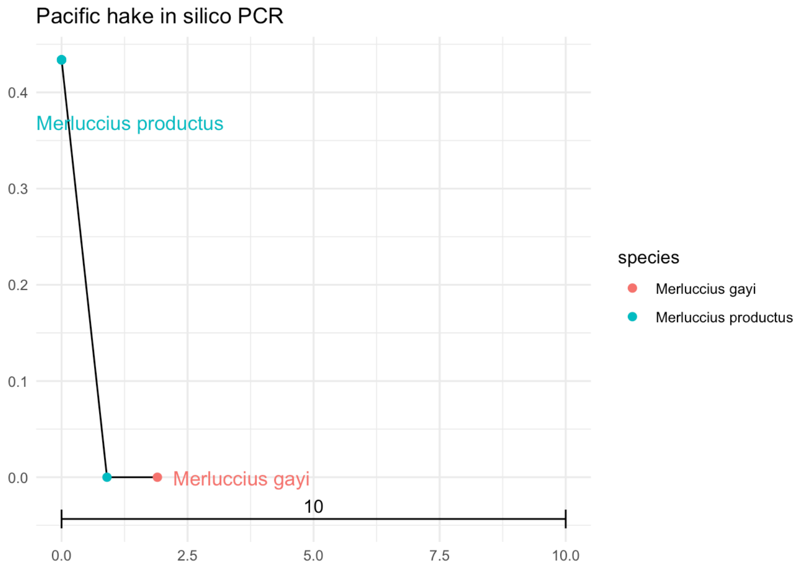

Pacific eulachon

```
## # A tibble: 2 x 2
## # Groups:   species [2]
##   species                                n
##   <chr>                              <int>
## 1 Dischistodus pseudochrysopoecilus      3
```

```
## 2 Thaleichthys pacificus            23
```

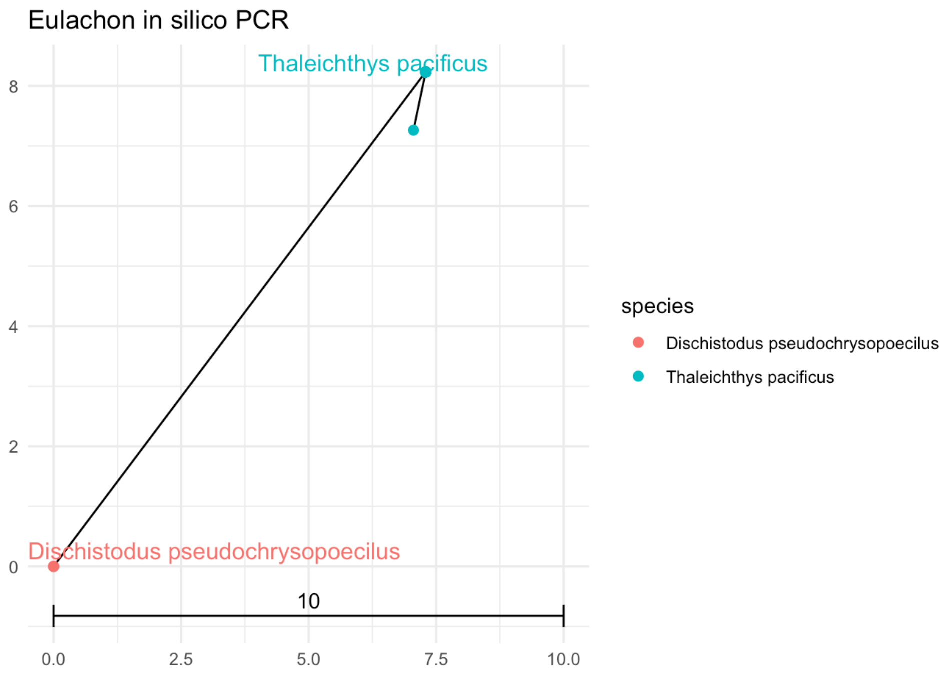

References

Cannon, M.V., Hester, J., Shalkhauser, A., Chan, E.R., Logue, K., Small, S.T. & Serre, D. (2016) In silico assessment of primers for eDNA studies using PrimerTree and application to characterize the biodiversity surrounding the Cuyahoga River. *Scientific Reports,* **6**.

de Blois, S. (2020) The 2019 Joint U . S .– Canada Integrated Ecosystem and Pacific Hake Acoustic-Trawl Survey: Cruise Report SH-19-06.

Hunter, M.E., Ferrante, J.A., Meigs-Friend, G. & Ulmer, A. (2019) Improving eDNA yield and inhibitor reduction through increased water volumes and multi-filter isolation techniques. *Scientific Reports,* **9,** 5259-5259.

Lu, J., Johnston, A., Berichon, P., Ru, K.L., Korbie, D. & Trau, M. (2017) PrimerSuite: A high-throughput web-based primer design program for multiplex bisulfite PCR. *Scientific Reports,* **7,** 41328-41328.

Mukhopadhyay, T. & Roth, J.A. (1993) Silicone lubricant enhances recovery of nucleic acids after phenol-chloroform extraction. *Nucleic Acids Research,* **21,** 781-782.
