# Supplementary material for: A workflow for the relative quantification of multiple fish species from oceanic water samples using environmental DNA (eDNA) to support large-scale fishery surveys
Source: PLoS One. 2021 Sep 27;16(9):e0257773. doi: 10.1371/journal.pone.0257773 (PMC8476043; doi:10.1371/journal.pone.0257773)
Supplement: S3 File — Complete collection, DNA extraction and quantification protocols. (PDF) [file pone.0257773.s003.pdf]

A workflow for the relative quantification of multiple fish species from oceanic water samples using environmental DNA (eDNA) to support large-scale fishery surveys

Ana Ramón-Laca, Abigail Wells, Linda Park

Supplemental material S3 – Complete workflow and protocols

1. Protocol for collection and preservation of eDNA
2. Protocol for DNA extraction: Phenol-chloroform-isoamyl alcohol DNA purification
3. Protocol for eDNA quantification by qPCR

Aim: to collect and filter 2.5 L of water at each depth from each CTD cast and preserved the filter at room temperature.

1. Protocol for collection and preservation of eDNA

Checklist of supplies:

- ☐ DNA away
- ☐ Kimwipes
- ☐ gloves
- ☐ Whirlpack bags (2.5 L is at 9.5" of the bag from the bottom)
- ☐ 2L pitchers (for whirlpack bags stability)
- ☐ utensils for getting membrane from filter (i.e. forceps)
- ☐ 500 ml filter cups with rubber stopper fitted with adapter for filter cup
- ☐ mixed cellulose ester sterile filters (1 µm, 47 mm ∅) (Advantec® Cat. A100H047A)
- ☐ pump
- ☐ 3-port manifold
- ☐ tubing adaptors
- ☐ wastewater container carboy
- ☐ labeled tubes containing 2 ml of Longmires's buffer
- ☐ bleach (5%) and bleach bucket
- ☐ distilled water
- ☐ 2 buckets (for bleach and rinsing)
- ☐ mesh bags
- ☐ drying racks
- ☐ bungees
- ☐ absorbent towels
- ☐ distilled water from the evaporator onboard and from the lab

Filter cups cleaning and assemblage:

1. Set up wash station (two buckets, one with 0.5% bleach, one with clean distilled H_2O)
2. Replace bleach and water stations at regular intervals (at least every other day, ≥0.5% bleach = ≥1.2L bleach in 20L bucket)
3. Place all used small items in a small mesh bag and the filter cups in a large mesh bag in bleach for at least 30 minutes
4. Rinse the abovementioned items for at least 10 minutes
5. Allow to dry on the drying racks
6. Wearing a fresh pair of gloves, mount filter cups with new sterile filter

Collection of water from Niskin bottles: Once the rosette has been brought back to the surface, water needs to be extracted from each Niskin bottle and transported into the lab for filtration. Each Niskin contains 10L of seawater. We want to collect 2.5L of water from each Niskin.

1. Let the water from the hull ("surface water sample") run for > 3 min while the CTD is being deployed
2. Collect 2 samples of 2.5 L each of "surface water" for each CTD cast
3. Wipe the spigot on the Niskin with DNAway to remove potential contaminants on the outside of the Niskin
4. Flush water (count to 10) from the Niskin to further reduce risk of contamination and collect 2.5 L of water in a whirlpack bag and place in 2L pitcher
5. Include a sampling negative control by collecting 2.5L of distilled H_2O (at least daily)

Filtration of water samples: Only DNA-free forceps should touch membrane! Water should be filtered immediately after collection.

1. Wipe working area with DNAway
2. Switch on the pump and set vacuum in the middle range between-8 and -12 bars (keep an eye on the gauge & adjust if necessary)
3. Pour water from whirlpack bag (supported by pitcher) into the filter cup, repeat until finished (2.5L of water)
4. Using DNA-free forceps fold filter and place in pre-labeled tube pre-filled with preservative buffer (2ml, Longmire Buffer in a 5 ml LoBind tube).
5. Filter 2L of ship distilled H_2O every time the cleaning distilled H_2O is replaced as a sampling negative control
6. Every 2-3 days 2L of lab diH_2O should be filtered as sampling negative controls
7. Note tube label in notes. Also note time filtered, membrane type, place filtered (field/lab, which lab), etc.
8. Store samples at room temperature away from UV light

Lysis buffer recipe (Longmire et al 1997):
To make 1 liter add in numerical order:
1. 975 ml double-distilled water
2. 100 ml of 1 M Tris-HCL, pH 8.0
3. 200 ml of 0.5 M EDTA, pH8.0

4. 2 ml of 5 M NaCl
5. 25 ml of 20% SDS (w/v)

Filter the buffer with an autofil PES bottle top filtration device (sterile 500ml, 0.22 μm) or autoclave after step 4 and then add the SDS.

2. Protocol for DNA extraction: Phenol-chloroform-isoamyl alcohol DNA purification

Checklist of supplies:

- ☐ proteinase k 20 mg ml^{-1}
- ☐ Dow corning high vacuum grease 5.3 oz
- ☐ Phenol-chloroform-isoamyl (25:24:1)
- ☐ Chloroform:isoamyl 24:1
- ☐ Isopropyl
- ☐ Ethanol (95%)
- ☐ 5 ml LoBind tubes Eppendorf
- ☐ Pipettes and tips (5 ml, 1 ml, 200 µl)
- ☐ 1.5 ml LoBind tubes Eppendorf
- ☐ Zymo One-Step inhibitor removal plates
- ☐ Incubator
- ☐ Centrifuge with a rotor with capacity for 5 ml tubes
- ☐ Tube racks (5 ml, 2 ml)
- ☐ Tube squeezing tool and sterile 15 ml syringes with wings or rings for grease dispensing
- ☐ Laboratory chemical fume hood or biosafety cabinet
- ☐ 5 M NaCl
- ☐ TlowE (Tris-HCl, EDTA, H_2O), [recipe at the end of document]

Step		Details
1	**heat-shock**	Filters in 2 ml of Longmire buffer at 95°C for 5' and then allow samples to cool to room temperature
2	**digestion**	add 100 µl proteinase K (final concentration 1 mg ml^{-1})
3	**incubation**	56°C at 120 rpm for 2h
4	**phase lock set up**	add ca. 800 µl of vacuum grease with a syringe onto the wall of the tube of the 5 ml tube
5	**PCI (25:24:1)**	add 2 ml of phenol-chloroform-isoamyl (25:24:1) pH 8
6	**centrifugation**	shake well and spin 13.3 x g for 5' at 4°C
7	**phase lock set up**	place ca. 800 µl of vacuum grease in 2 sets of empty tubes for CI. (These can be prefilled for convenience)
8	**CI**	add 2.2 ml of chloroform:isoamyl 24:1 and decant aqueous layer from step 6. **Important**: only add the chloroform just before use (while the tubes with PCI are in the centrifuge) so it does not affect the phase lock if grease is at the bottom.
9	**centrifugation**	shake and spin 13.3 x g for 5' at 4°C
10	**CI**	add 2.2 ml of chloroform:isoamyl 24:1 and decant aqueous layer from step 9
11	**centrifugation**	shake and spin 13.3 x g for 5' at 4°C
12	**Isopropanol**	add 2 ml of isopropanol (can be prefilled for convenience), add 80 µl of 5M NaCl and decant aqueous layer from step 11
13	**mixing**	invert several times
14	**precipitation**	overnight (or 2 h) at room temperature
15	**centrifugation**	spin 13.3 x g for 30' at 4°C
16	**wash × 2**	pour liquid off slowly and add 800 µl of ice cold 70% EtOH
17	**centrifugation**	shake and spin 13.3 x g for 5' at 4°C **and repeat wash**
18	**drying**	pour liquid off slowly and allow tubes to dry for 1 h or until dry
19	**resuspension**	once they are dry, resuspend in 100 µl TE buffer (warm - 37°C)
20	**storage**	store in the freezer (-20-80 °C)

3. Protocol for eDNA quantification by qPCR

Checklist of supplies:

- ☐ Electronic four-channel Adjustable 10-300 µl, from 5 ml to 96 well plate pipette for plating eDNA in low binding 96 well plates
- ☐ Electronic eight-channel Adjustable 10-300 µl, from 1.5 ml to 96 well plate pipette (Integra) - to transfer the standards into a plate
- ☐ Electronic eight-channel Adjustable 1-10 µl, from 96 well to 384 well pipette
- ☐ 96 x 300 µL tips box
- ☐ 384 x 10 µL tips box
- ☐ TaqPath ProAmp Multiplex Master Mix (uses Mustang Purple as passive reference)*
- ☐ Primer and TaqMan probes
- ☐ gBlocks
- ☐ IPC at 1000 copies $µl^{-1}$
- ☐ LoBind 96-well plates
- ☐ LoBind 1.5 tubes
- ☐ 384 well PCR plates
- ☐ optical seals and sealing paddle
- ☐ Electronic pipette 5 µl - 100 µL (programed to repeat pipette 8 µl)
- ☐ 384 Well Aluminum cold block
- ☐ eDNA for questioned samples
- ☐ 1 M Tris-HCL, pH 8.0
- ☐ 0.5 M EDTA, pH8.0

*This allows for four channels to be used for species detection and IPC. We have successfully used FAM (MGB group + NFQ), VIC (MGB group + NFQ), ABY (QSY) and JUN (QSY)

<u>Prepare template DNA</u>

1. Pipetting the template into the 384 well qPCR plate is most efficiently done from a 96 well PCR plate with the electronic multichannel adjustable pipette. Make sure you have enough DNA for triplicate runs (keep on cold block with temporary seal if possible)

<u>Prepare Standard Curve DNA</u>

2. Standard should be made fresh in 1.5 ml low retention Eppendorf centrifuge tubes every week starting from an already made aliquot of the gBlocks at 1 x 10^6 copies $µl^{-1}$ stored in the freezer. Keep in the refrigerator and plate them daily into a low retention plate. Use as many times as necessary each day. Also have some TlowE plated to be dispensed in the ntc.
3. Standards should be made by adding 100 µl of the previous standard into 900 µL of TlowE
4. Standards should be included at least in triplicate (<u>six replicates for the lowest</u>) at concentrations:
    - 1 x 10^5 copies $µl^{-1}$ x 3
    - 1 x 10^4 copies $µl^{-1}$ x 3
    - 1 x 10^3 copies $µl^{-1}$ x 3
    - 1 x 10^2 copies $µl^{-1}$ x 3
    - 1 x 10^1 copies $µl^{-1}$ x 3
    - 5 copies $µl^{-1}$ x 3
    - 1 copy $µl^{-1}$ x 6
5. Also include at least three non-template controls that will have the IPC included and add 2 µL of TlowE to each
6. Set up qPCR assay

7. Create Master mix with 2×TaqPath ProAmp Multiplex Master mix, primer mix and probe mix, IPC and water (see below)
8. Use pipettor to aliquot master mix into 384 well plate (8 µl per well) making sure the plate is cold to avoid evaporation and changes in concentration)
9. Use electronic multichannel adjustable 1-10 µl pipette to distribute DNA into wells. Discard excess DNA and tips between. Having the right number of tips already set apart is recommended to orientate yourself in the 384 well plate
10. Set optical sheet onto 384 well plate and seal tape to plate using the paddle
11. Spin plate to displace bubbles
12. Run set up on the qPCR from a template

TlowE:

To make 50 ml

1. 500 µl of 1 M Tris-HCL, pH 8.0

2. 10 µl of 0.5 M EDTA, pH8.0

3. up to 50 ml of nuclease free H_2O

PCR conditions and master mix:

1 cycle 10' at 95°C
45 cycles 15''at 95°C and 1' at 60°C

5 µl of TaqPath ProAmp Multiplex Master Mix
0.9 µM of forward and reverse primers (in a mix)
0.2 µM of TaqMan probes (in a mix)
1 µl of IPC at 1000 copies μl^{-1}
0.9 µl of H_2O
2 µl of DNA/gBlocks mix or TlowE (eDNA/standards or ntc)
